# Application of hierarchical oligonucleotide primer extension (HOPE) to assess relative abundances of ammonia- and nitrite-oxidizing bacteria

**DOI:** 10.1186/s12866-017-0998-2

**Published:** 2017-04-04

**Authors:** Giantommaso Scarascia, Hong Cheng, Moustapha Harb, Pei-Ying Hong

**Affiliations:** Biological and Environmental Science & Engineering Division (BESE), King Abdullah University of Science and Technology (KAUST), Water Desalination and Reuse Center (WDRC), Thuwal, 23955-6900 Saudi Arabia

**Keywords:** Single nucleotide primer extension, Quantitative monitoring, 16S rRNA gene-based amplicon sequencing, AOB/NOB ratio, Shock loading event

## Abstract

**Background:**

Establishing an optimal proportion of nitrifying microbial populations, including ammonia-oxidizing bacteria (AOB), nitrite-oxidizing bacteria (NOB), complete nitrite oxidizers (comammox) and ammonia-oxidizing archaea (AOA), is important for ensuring the efficiency of nitrification in water treatment systems. Hierarchical oligonucleotide primer extension (HOPE), previously developed to rapidly quantify relative abundances of specific microbial groups of interest, was applied in this study to track the abundances of the important nitrifying bacterial populations.

**Results:**

The method was tested against biomass obtained from a laboratory-scale biofilm-based trickling reactor, and the findings were validated against those obtained by 16S rRNA gene-based amplicon sequencing. Our findings indicated a good correlation between the relative abundance of nitrifying bacterial populations obtained using both HOPE and amplicon sequencing. HOPE showed a significant increase in the relative abundance of AOB, specifically *Nitrosomonas*, with increasing ammonium content and shock loading (*p* < 0.001). In contrast, *Nitrosospira* remained stable in its relative abundance against the total community throughout the operational phases. There was a corresponding significant decrease in the relative abundance of NOB, specifically *Nitrospira* and those affiliated to comammox, during the shock loading. Based on the relative abundance of AOB and NOB (including commamox) obtained from HOPE, it was determined that the optimal ratio of AOB against NOB ranged from 0.2 to 2.5 during stable reactor performance.

**Conclusions:**

Overall, the HOPE method was developed and validated against 16S rRNA gene-based amplicon sequencing for the purpose of performing simultaneous monitoring of relative abundance of nitrifying populations. Quantitative measurements of these nitrifying populations obtained via HOPE would be indicative of reactor performance and nitrification functionality.

**Electronic supplementary material:**

The online version of this article (doi:10.1186/s12866-017-0998-2) contains supplementary material, which is available to authorized users.

## Background

The conventional process of removing nitrogen from municipal wastewater streams involves nitrification followed by denitrification. Nitrification in the wastewater treatment process is generally performed in two consecutive steps. The first step is ammonia oxidation by either ammonia-oxidizing bacteria (AOB) or ammonia-oxidizing archaea (AOA), and the second step is nitrite oxidation by nitrite-oxidizing bacteria (NOB). Representative AOB genera include *Nitrosomonas* and *Nitrosospira* while *Nitrososphaera* and *Nitrosopumilus* would account as two examples of AOA. Representative NOB include *Nitrospira* and *Nitrobacter*. In recent years, three members within the genus *Nitrospira*, namely Candidatus *Nitrospira inopinata*, Candidatus *Nitrospira nitrosa* and Candidatus *Nitrospira nitrificans*, were shown to perform complete nitrification in a single step and are referred to as comammox [1, 2]. Although comammox are not yet isolated from municipal wastewater treatment plants (WWTP) and their contribution in terms of full nitrification in such systems remain unknown, comammox had been shown to use urea as ammonium source for nitrification and that this trait should in theory enable them to thrive in WWTP environments where urea is often present [1].

The commensal interaction between AOB/AOA and NOB, and the potential importance of *Nitrospira*-affiliated comammox suggest that the nitrification process can be prone to failure when there is a suboptimal ratio of AOB/AOA to NOB, particularly that of *Nitrospira*. This is primarily due to the effect of hydroxylamine, an intermediate of the conversion of ammonia to nitrite, which has a strong inhibitory effect on nitrite oxidizers when released by AOB/AOA [3]. Failure of the nitrification system in a WWTP can result in discharge of effluent that contains total nitrogen (TN) higher than the permissible limit (< 15 mg/L in Saudi Arabia). This can result in potentially detrimental environmental impacts in the form of eutrophication of the receiving water bodies. Hence, establishing an optimal ratio and abundance of AOB/AOA to NOB is important in ensuring the functionality of the nitrification process so that treated effluent will meet the discharge limits for TN.

In such instance, a quantitative method capable of efficiently targeting AOB/AOA and NOB relative abundances would be particularly useful in tracking the proportional ratio between these two groups. Various methods like quantitative PCR (qPCR) and fluorescent in-situ hybridization (FISH) were used to directly quantify the abundances of AOB and NOB. Through these approaches, it was determined that *Nitrosomonas* likely accounts for the largest fraction of AOB in conventional activated sludge systems. Additionally, in conventional municipal wastewater streams in which ammonium concentrations ranged from 12 to 50 mg/L, *Nitrospira* was the predominant NOB while *Nitrobacter* was generally in low abundance [3–5].

Although qPCR and FISH have been routinely used in earlier studies, both approaches have substantial limitations. For example, qPCR requires standard curves to be established for individual targets. New generation of PCR (i.e., digital PCR, dPCR) does not require standard curves to be generated but similar to conventional qPCR, remains limited by the number of fluorophores available in the ultraviolet spectrum. Hence, both quantitative PCR approaches have limited multiplexing throughput capability. Furthermore, both qPCR approaches can be prone to non-specific amplification when SYBR® green fluorescence reporters are utilized, and would require an additional melting curve analysis or verification step to differentiate between true-positive and false-positive amplifications. Although Taqman® probes can be developed for use in qPCR and dPCR, such probes are relatively expensive and remain challenging to design for a specific microbial target within the internal region of two flanking qPCR primers. Digital PCR being a considerably new technology, also remains rather costly till this date compared to traditional qPCR. FISH, on the other hand, can be affected by the in-situ accessibility of labeled probes through the cell membranes and the copy numbers of rRNA genes inside AOB and NOB. For example, species of *Nitrobacter*, *Nitrosomonas*, *Nitrosospira* and *Nitrospira* are reported to have one copy of 16S rRNA gene per cell. This copy number of 16S rRNA genes per genome is lower than the mean copy number of 4.12 among the 2865 microbial genomes currently available in the same Ribosomal RNA Operon Copy Number database [6].

Hierarchical oligonucleotide primer extension (HOPE) was developed as a method to complement other quantitative methods like qPCR and FISH by providing a high-throughput multiplexing platform capable of addressing some of the previous methods’ limitations. In this method, oligonucleotide primers are designed to target genes, usually 16S rRNA genes, at different hierarchical taxonomical levels (from domain to species). In the presence of a DNA template, the primers anneal at the designed targeting position of the 16S rRNA gene and extend with a single fluorescently labeled dideoxynucleoside triphosphate (ddNTP). The extended primers can then be identified on a DNA sequencer based on their anticipated fragment size and fluorescence. The peak areas of the individual fragments can then be used to determine the relative abundances of bacterial targets of interest [7].

The HOPE method has been developed to detect specific bacterial populations of interest including host-associated Bacteroidales and *Bifidobacterium*, cyanobacteria, and methanogens [8–11]. In these studies, HOPE has been demonstrated to multiplex up to 10 primers in a single reaction, allowing for the evaluation of a range of relevant bacterial indicators in a time-efficient manner [12]. The high-throughput capability of HOPE stems from the use of different fragment sizes of primers and fluorescence colors to distinguish between extended primers. To exemplify, most primers are synthesized with a length of 18 to 25 nucleotides (nt) while most DNA sequencer platforms are able to detect up to four different fluorescence colors. Hence, in theory, the combination of these two differentiating factors would mean that HOPE is capable of multiplex up to 32 primers in one single reaction tube. Aside from its multiplexing capability, it was demonstrated that HOPE has a detection limit of 0.1% of the total PCR-amplified bacterial targets, and that the entire duration required to carry out a single HOPE reaction and to analyze for the extended primer on a DNA sequencer is <1 h, which would be comparable to the time needed to carry out qPCR [12].

Given the advantages of HOPE, the method is a potentially useful tool in tracking the relative abundance of nitrifying populations. In the present study, we applied the HOPE approach to target groups of AOB and NOB (including *Nitrospira*-affiliated comammox) in wastewater environments. The method was tested against samples collected from a lab-scale biofilm-based trickling bioreactor. The total microbial community, including the nitrifying groups, was analyzed by 16S rRNA gene-based high-throughput sequencing for comparison against the HOPE data. To further demonstrate the use of HOPE in monitoring these relative abundances, the trickling bioreactor was subsequently challenged with high concentrations of incoming ammonium content so as to simulate a toxic shock event that could potentially crash the nitrification system. The changes in AOB and NOB proportions were then tracked by the HOPE approach and correlated to reactor performance. Wastewater samples from a fully operational wastewater treatment plant (WWTP) were also analyzed in order to further validate the method.

## Methods

### HOPE primer layout

HOPE was performed with a total of 13 primers targeting ammonia-oxidizing genera *Nitrosomonas* and *Nitrosospira* as well as nitrite-oxidizing genera *Nitrospira* and *Nitrobacter* (Table 1). Primers were sourced from the probeBase database [13]. The mismatch positions of non-targets were moved to the 3′-end of the HOPE primers as it was previously found that mismatches located at the 3′-end of the HOPE primers would not facilitate nucleotide base extension of primers, and would hence lead to improved specificity [14]. Re-positioning of the mismatches result in minimal change to the original coverage of the targeted bacterial groups. The coverage of the primers was then verified in-silico against the Ribosomal Database Project (RDP) database [15], and shown in Additional file 1. Primers were arranged into four tubes, with either 27F or 338F primer included in each tube to provide a normalization of the abundance of each nitrifying bacterial target against the total bacteria. To differentiate primers that extend with the same ddNTP and hence color during capillary electrophoresis, some primers were modified with a polyA tail at the 5′-end of the primer (Table 1). Primers in each tube were prepared to form a final concentration of 10 μM.

**Table 1:** HOPE primers used to target the different ammonia- and nitrite-oxidizing bacterial groups

	Target	Primer sequence (5'-3')	Extended ddNTP	PolyA-tail (nt)	Reference
NOB tube 1: *Nitrospira*					
27F	Most Bacteria	AGAGTTTGATCCTGGCTCAG	A	0	[38]
Ntspa712	4451 out of 8171 members of Nitrospirae; also targets comammox bacteria Candidatus *N. inopinata*	CGCCTTCGCCACCGGCCTTCC	T	0	[39]
Ntspa572	1835 out of 8171 members of Nitrospirae; also targets comammox bacteria Candidatus *N. inopinata*	AACCGCCTACGCTCCCTG	T	0	This study
Ntspa1429	107 out of 8171 members of Nitrospirae; *Nitrospira* cluster II	TGGCTTGGGCGACTTCAG	G	6	Modified from [37]
NOB tube 2: *Nitrobacter*					
Eub338Ia	Most Bacteria	GCTGCCTCCCGTAGGAG	T	8	[12]
Nit1017	107 out of 195 genus *Nitrobacter*	TGC TCC GAA GAG AAG GTC ACA	T	6	Modified from [40]
Nit1000	117 out of 195 genus *Nitrobacter*	TGC GAC CGG TCA TGG	A	0	[41]
AOB tube 1: *Nitrosomonas* and *Nitrosospira*					
27F	Most Bacteria	AGAGTTTGATCCTGGCTCAG	A	0	[38]
NmoCL6a	379 out of 7530 members of order Nitrosomonadales, *Nitrosomonas* cluster 6a; mainly targeting unclassified Nitrosomonadaceae	AAGCATAAGGTCTTTCGATCCCCT	G	3	Modified from [42]
NmoCL6b	1749 out of 7530 members of order Nitrosomonadales, *Nitrosomonas* cluster 6b; Major coverage also includes other non-AOB and NOB groups	GGATCAGGCTTGCGCCC	A	0	[42]
AOB tube 2: *Nitrosomonas* and *Nitrosospira*					
Eub338Ia	Most Bacteria	GCTGCCTCCCGTAGGAG	T	8	[12]
Nso190	1295 out of 7530 members of order Nitrosomonadales	CGATCCCCTGCTTTTCTC	C	9	[41]
Nmo218	321 out of 897 members of unclassified Nitrosomonadaceae	CGGCCGCTCCAAAAGCAT	A	0	[43]
Nse1472	208 out of 911 members of genus *Nitrosomonas*	ACCCCAGTCATGACCCCC	A	6	[44]
Nsv443	934 out of 5714 members of genus *Nitrosospira*	CCGTGACCGTTTCGTTCCGGC	T	0	[41]

Target coverage for each primer was identified using the RDP Probe match function

### HOPE reference template preparation

To prepare the HOPE reference templates for nitrifying bacterial populations, extracted DNA from a separate trickling nitrification biofilter that was set up in year 2007 was first amplified with 27F (5′-AGA GTT TGA TCC TGG CTC AG-3′) to 1492R (5′-GGY TAC CTT GTT ACG ACT T-3′) primers and purified for use in 16S rRNA gene clone library construction. Approximately 100 clones were picked and sequenced to identify nitrifying bacterial species. Two clones associated with Candidatus *Nitrospira defluvii* and *Nitrosomonas eutropha* were obtained and used as the reference template for HOPE primers Ntspa712, Ntspa1429, Nso1225b and Nse1472. The reference standards for Ntspa572, Nit1017, NmoCL6a, NmoCL6b and NspCL3 were obtained by first amplifying the 16S rRNA gene portion targeted by that respective HOPE primer coupled with 27F. The reference standards for primers Nmo218 and NspCL1 were obtained by amplifying the 16S rRNA gene portion with a sense-strand version of that respective HOPE primer and 1492R. All amplicons were gel-excised, purified and submitted for Sanger sequencing at the KAUST Genomics Core lab. Sequences were checked to ensure perfect matches in regions targeted by the specific primers. Reference templates for Nit1000 and Nso190 were obtained by checking the above-mentioned reference templates for perfect match regions. Amplicons with regions that perfectly matched the targeted primers were then individually ligated into the pCR2.1 vector using the TA cloning kit (Thermo Fisher Scientific, Carlsbad, CA). The ligated vectors were individually transformed into TOP10 competent cells, and transformed cells were then extracted for the plasmid using PureYield plasmid miniprep system (Promega, Madison, WI). The insert gene was resequenced by the Sanger approach to verify for perfect match regions. Subsequently, plasmid DNA concentrations were measured using an Invitrogen Qubit 2.0 fluorometer (Thermo Fisher Scientific, Carlsbad, CA) and diluted to 10 ng/μL to be used as HOPE standards in determination of calibration factors. Calibration factors are normalization factors to account for differences in peak areas between two extended HOPE primers [8], and are calculated based on Eq. (1) below:

1 $$ {CF}_{A- B}=\frac{{\mathrm{Peak}\ \mathrm{area}\ \mathrm{of}\ \mathrm{extended}\ \mathrm{primer}\ \mathrm{A},\mathrm{P}}_{\mathrm{a}}}{{\mathrm{Peak}\ \mathrm{area}\ \mathrm{of}\ \mathrm{extended}\ \mathrm{primer}\ \mathrm{B},\mathrm{P}}_{\mathrm{b}}} $$

where primer B is targeting at a higher hierarchical level compared to primer A.

### HOPE reactions and capillary electrophoresis

For each sample that was to be tested with HOPE, the bacterial 16S rRNA genes were first amplified using 27F coupled with 1492R primer in PCR amplifications comprising of 5 ng DNA, 25 μL of Epicentre Biotechnologies FailSafe Premix F (Illumina, Madison, WI), 200 nM (each) of forward and reverse primers, 0.5 U of ExTaq DNA polymerase (Takara, Japan), and molecular-biology grade water as necessary to reach a total volume of 50 μL. PCR was performed with 1 cycle of 95 °C for 30 s followed by 25 cycles of thermal cycling (denaturation at 95 °C for 30 s; annealing at 55 °C for 45 s; extension at 72 °C for 60 s) and a final cycle of 72 °C for 10 min. All amplicons were purified using Wizard SV Gel and PCR Clean-up kit (Promega, Madison, WI), measured for their concentrations, and diluted to 10 ng/μL to be used as templates in HOPE reactions. Each HOPE standard and sample reaction was carried out in a reaction volume that was comprised of 2.5 μL of Applied Biosystems SNaPshot multiplex kit (Thermo Fisher Scientific, CA), 0.5 μL of 10 μM primer mix, 0.5 μL of DNA template and 1.5 μL of molecular-biology grade water. The HOPE thermal cycling program consisted of 20 cycles of denaturation (96 °C for 10 s), annealing (60 °C for 30 s) and extension (72 °C for 15 s). After the primer extension reaction, 5 μL of 200 U recombinant shrimp alkaline phosphatase, rSAP (New England Biolabs, Ipswich, MA), was added to each reaction and the mixture was incubated at 37 °C for 60 min prior to denaturation at 80 °C for 10 min. The rSAP-digested samples were prepared for capillary electrophoresis by first adding 1 μL of the sample to 12 μL of Applied Biosystems Hi-Di formamide and 0.3 μL of GeneScan 120 LIZ size standard, and the mixed samples were denatured at 96 °C for 5 min prior to capillary injection in the Applied Biosystems 3500 Series genetic analyzer. Extended primers were identified on GeneMapper v 4.1 based on the extended nucleotide base (i.e., color) and fragment size. Peak areas of the primers extended in the presence of reference standards were recorded for determination of calibration factors as described previously [8] and in Eq. (1). The same was performed for the primers extended in the presence of template obtained from tested samples. The relative abundance of 16S rRNA gene amplicons targeted by primer A with respect to those targeted by primer B can then be calculated based on Eq. (2) below:

2 $$ \mathrm{Relative}\ \mathrm{abundance}\ \mathrm{of}\ \mathrm{target}\ \left(\%\right)\kern0.75em =\kern1em \frac{\mathrm{Peak}\ \mathrm{area}\ \mathrm{of}\ \mathrm{primer}\ \mathrm{A}}{\mathrm{Peak}\ \mathrm{area}\ \mathrm{of}\ \mathrm{primer}\ \mathrm{B}\times \kern0.5em {CF}_{A- B}}\times 100\% $$

where primer B is targeting at a higher hierarchical level compared to primer A.

### 16S rRNA gene-based high-throughput sequencing and data analysis

For all samples quantified by HOPE, Illumina MiSeq amplicon sequencing was performed to validate the HOPE data and to provide information on the total microbial community. To prepare the 16S rRNA gene amplicon libraries, 515F (5′- Illumina overhang- GTG YCA GCM GCC GCG GTA A- 3′) and 907R (5′- Illumina overhang- CCC CGY CAA TTC MTT TRA GT- 3′) primers were modified to encode the overhang adaptor sequences, and used to amplify the 16S rRNA genes. The thermal cycling program included an initial denaturation stage at 95 °C for 3 min, followed by 25 cycles of denaturation at 95 °C for 30 s, annealing at 55 °C for 30 s and extension at 72 °C for 30 s, and then a final extension stage at 72 °C for 5 min. PCR amplicons were then purified by AMPure XP beads (Beckman Coulter, CA) prior to the index PCR. Nextera XT Index (Illumina, San Diego, CA) was incorporated into each of the individual samples during PCR. The thermal cycling program included a denaturation stage at 95 °C for 3 min, followed by 8 cycles of denaturation at 95 °C for 30 s, annealing at 55 °C for 30 s and extension at 72 °C for 30 s, and then a final extension stage at 72 °C for 5 min. The final indexed PCR amplicons were again purified by AMPure XP beads and quantified for the concentrations using an Invitrogen Qubit 2.0 fluorometer. The controls for all PCR reactions were negative for amplification. Purified amplicons were submitted to the KAUST Genomics Core lab for unidirectional sequencing on an Illumina MiSeq platform. Raw sequence reads were handled using procedures described previously [16]. To annotate the 16S rRNA gene sequences obtained from high-throughput sequencing, RDP Classifier was used for taxonomical assignments at a 95% confidence level [17].

### Primer-E analysis and statistical tests

The taxonomical assignment for each sample obtained by high-throughput amplicon sequencing was calculated for relative abundances of the individual bacterial and archaeal genera. Special emphasis was made to collate the relative abundance of the *Nitrospira*, *Nitrosomonas*, *Nitrobacter* and *Nitrosospira* so as to provide validation against the relative abundances of these genera obtained by HOPE method. In addition, the relative abundance of all taxonomical assignments obtained by MiSeq amplicon sequencing were collated alongside the relative abundances of the bacterial targets identified by HOPE. Both datasets were individually input into Primer E version 7 [18], square-root transformed, and then computed for their Bray-Curtis similarities. The Bray-Curtis distance matrix was used for multivariate analysis on a non-metric threshold multidimensional scaling (nMDS) plot. The nMDS plots utilizes a distance matrix applied to all samples by which each is represented as a point in the two-dimensional space. The x and y-axes of the nMDS plot do not represent any parameters. Instead, the distance between the positioning of two samples would denote the extent of similarity between these two samples. To illustrate, samples with higher similarity are closer in proximity in the nMDS and vice versa. The stress value measures the scatter in the nMDS plot, and ranges from 0 to 1, with 0 denoting a good representation of the positioning of the samples in the two-dimensional space shown in the nMDS plot and vice versa. Bacterial targets that exhibited >0.7 correlation with the multivariate patterns on the nMDS were overlaid as vectors. Vectors are directional lines emanating from a common origin, extending in the directions in which the marked bacterial targets increase in relative abundance, and hence play a dominant role in the subclustering of samples on the nMDS plot. All other significance tests were conducted using two-tailed t-tests on Microsoft Excel 2013.

### Biofilm reactor setup and operation

**Fig. 1:**
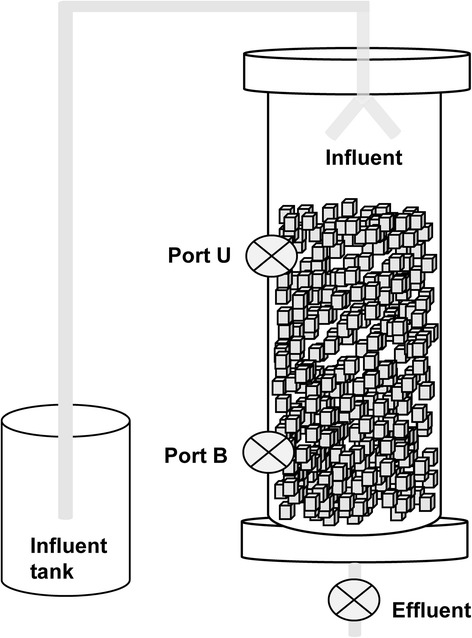
Trickling nitrification biofilm reactor column. The reactor was operated through six phases A through E, with increasing ammonium (NH_4_^+^) concentration in the influent during phase A through E and a decrease to 150 mg/L NH_4_^+^ in the influent at phase D2. Samples were collected at the top (U) and bottom (B) locations from Port U and B, respectively. Influent and effluent were also collected from the marked sampling ports

The samples that were quantified for the respective nitrifying populations by HOPE and 16S rRNA gene-based amplicon sequencing were obtained from a trickling biofilm nitrification reactor. The trickling biofilm reactor was first packed with sponge cubes, each of dimension 1 by 1.5 cm, to a total volume of 4.5 L (Fig. 1). 500 mL of sludge collected from the aerobic activated sludge tank of the full scale WWTP was inoculated upon commencement of operation and another 500 mL of aerobic sludge was added 2 weeks later. During the startup phase (Phase A, day 0 to 39), basal substrate containing 40 mg/L ammonium (NH_4_^+^-N) was fed into the reactor. The components per L of basal substrate were modified from a previous study [19] and include: 153 mg NH_4_Cl, 480 mg NaHCO_3_, 24 mg MgSO_4_.7H_2_O, 20 mg K_2_HPO_4_, 11.3 mg CaSO_4_.2H_2_O and 7.1 mg FeCl_3_.6H_2_O, 100 μL of trace elements that contained 0.274 g/L Na_2_MoO_4_.2H_2_O, 2.74 g/L ZnSO_4_.7H_2_O, 0.036 g/L CuSO_4_.5H_2_O, 0.03 g/L CoCl_2_.6H_2_O, 7 g/L FeSO_4_.7H_2_O and 1.55 g/L MnCl_2_.2H_2_O. Upon reaching stabilization and from day 40 onwards, the basal substrate was prepared by diluting the anaerobic effluent that was high in ammonium content [20]. The anaerobic effluent contained on average 160 mg/L NH_4_^+^-N and 40 mg/L of chemical oxygen demand (COD), and the ammonium content in the basal feed was diluted accordingly to provide 80 mg/L NH_4_^+^-N from day 40 to 83 (Phase B), 110 mg/L NH_4_^+^-N from day 84 to 120 (Phase C), and 150 mg/L NH_4_^+^-N from day 121 to 150 (Phase D1). Ammonium concentration was subsequently increased to 200 mg/L NH_4_^+^-N from day 151 to 179 (Phase E), and diluted to 150 mg/L NH_4_^+^-N from day 180 to 270 (Phase D2). Throughout operation, the pH of trickling biofilm reactor was maintained between 7.0 and 7.5 and aeration was provided at a sparging rate of 2 L/min.

### Biofilm reactor sampling and DNA extraction

Three sponge cubes were sampled individually from both the top and bottom sampling ports (Fig. 1), and referred to as U and B samples subsequently in this study. New replacement sponges were placed back into the reactor after each biomass sampling so as to provide new substrata for the adherent bacteria. Subsequent samplings were carried out such as to avoid sampling the replacement sponges. Sponges with the adherent biomass were placed in 50 mL centrifuge tubes, each containing 20 mL of 1X phosphate buffer saline (PBS). The samples were then vortexed at maximum speed for 10 min and the sponge cubes removed aseptically by sterile forceps. The biomass suspensions in the 50 mL tubes were then centrifuged at 6800×*g* for 10 min to collect cell pellets. 0.2 g of biomass from cell pellet was weighed and extracted for its DNA. DNA was extracted using the UltraClean Soil DNA Isolation Kit (MoBio Laboratories, Carlsbad, USA) with slight modifications to the protocol by adding lysozyme and achromopeptidase to the lysis buffer [21].

### Full-scale WWTP sampling and DNA extraction

Another set of samples that were quantified for the respective nitrifying populations by HOPE and 16S rRNA gene-based amplicon sequencing were sampled from a full-scale wastewater treatment plant located in KAUST. The WWTP is a full-scale membrane bioreactor (MBR) equipped with the following process units: (i) grid mesh screen, (ii) primary clarifier, (iii) anoxic-oxic activated sludge tanks, (iv) submerged membrane tank, and (v) holding tank for chlorination. Influent was collected after the primary clarifier, effluent was collected at the MBR discharge point and chlorinated effluent was collected from the holding tank. Activated sludge was collected in the oxic zone of the sludge tank. Sampling was performed on a monthly basis from July to December 2015, with the exception of October 2015 during which two sample sets were collected. Influent samples were prepared for DNA extraction by centrifuging 50 to 100 mL of influent at 10000×*g* for 20 min to obtain the biomass pellet. 2 L each of effluent and chlorinated effluent were individually filtered through 0.4 μm polycarbonate membranes to retain the biomass. Biomass samples were extracted for their DNA using the UltraClean Soil DNA Isolation Kit (MoBio Laboratories, Carlsbad, USA) with slight modifications to the protocol by adding lysozyme and achromopeptidase to the lysis buffer [21].

### Water quality measurement for biofilm reactor samplesand wastewater streams

Samples from the fresh basal substrate fed into the trickling biofilm reactor were collected as influent, and samples collected from the discharge port were collected as effluent (Fig. 1). Ammonium (NH_4_^+^-N), nitrite (NO_2_^-^-N), nitrate (NO_3_^-^-N) concentrations in influent and effluent samples (*n* = 75 each) collected every 2–3 d throughout the reactor operational period were measured using Test ‘N Tube high range ammonia kit, TNTplus 839, and TNTplus 835, respectively. COD was measured using either LCK 314 (15–150 mg/L) or LCK 514 COD (100–2000 mg/L) cuvette test vials depending on the concentration to be measured. All measurements were conducted based on protocols specified by the manufacturer (Hach-Lange, Manchester, UK). The water quality data obtained from the trickling biofilm reactor were collated, log-transformed and normalized within Primer-E version 7 [18]. The normalized data were then used to generate a principal component analysis (PCA) plot. Water quality parameters that exhibited >0.7 correlation with the multivariate pattern on the PCA were overlaid as vectors. For the wastewater samples obtained from the WWTP, nitrate concentrations in all three streams were measured in lab using the TNTplus 835. Total nitrogen in all streams as well as ammonia in the influents were measured and provided by the plant operator.

## Results

### AOB abundance in biofilm reactor quantified by HOPE throughout the operational phases

**Fig. 2:**
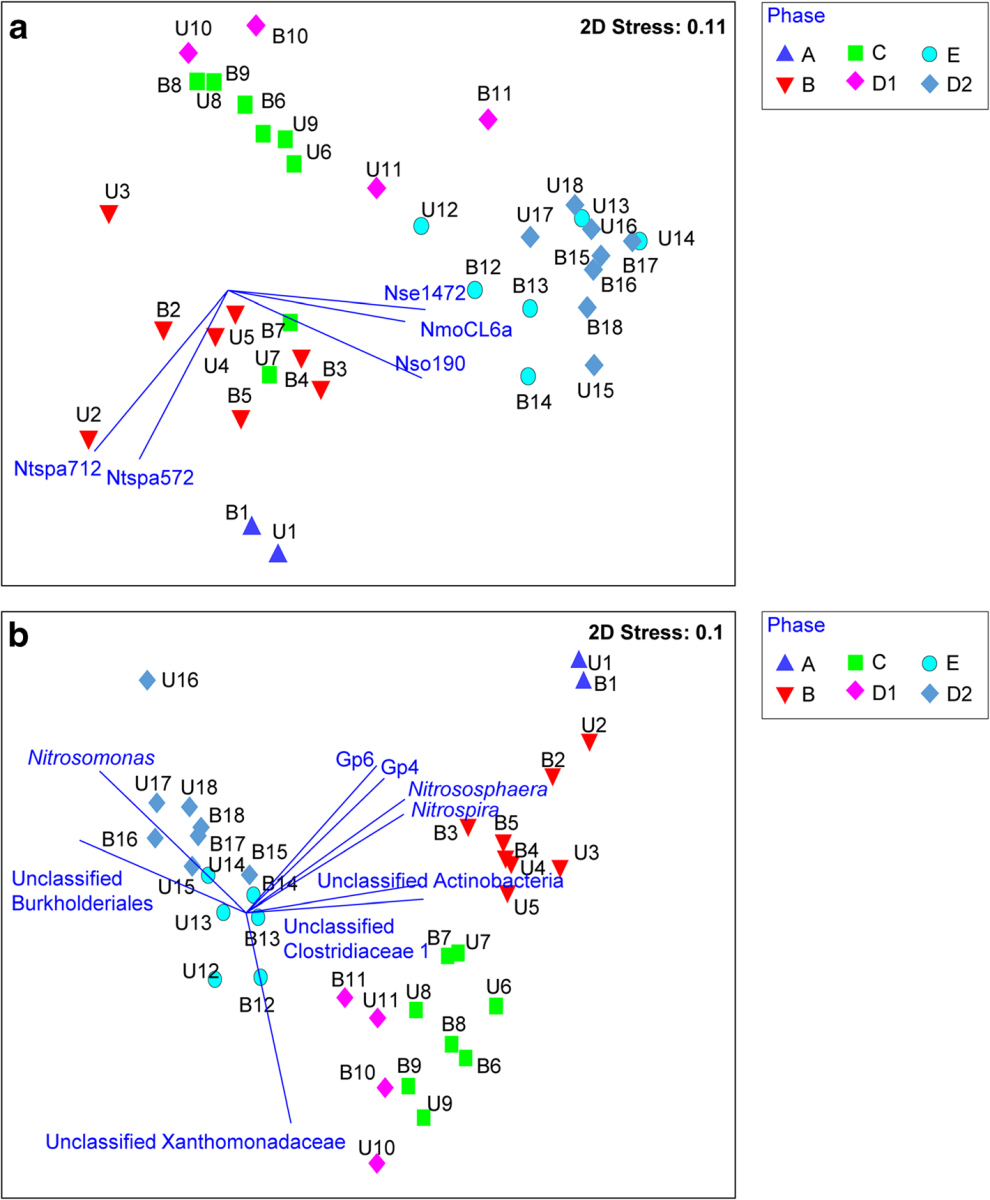
Multivariate analysis of the microbial data. The relative abundance of AOB and NOB targeted by HOPE primers revealed differences throughout the operational phases. **a**. The relative abundances of the total microbial community obtained from high-throughput sequencing also showed similar clustering based on the operational phases (**b**)

The relative abundances of nitrifying bacterial populations quantified by the HOPE approach were collated for multivariate analysis on a multidimensional scaling (nMDS) plot (Fig. 2a). The positioning of the samples on the nMDS plot showed that operational phase was the dominant factor accounting for the subclustering of samples. Vector-based analysis showed an increase in the abundance of AOB in Phases E and D2 compared to the early operational phases. To illustrate, the relative abundance of AOB groups, specifically *Nitrosomonas* targeted by Nse1472 and NmoCL6a, increased from 2.2 ± 1.8% and 3.4 ± 1.3% of total bacteria, respectively, in Phase B to 18.9 ± 10.3% and 26.5 ± 9.5% of total bacteria, respectively, in Phase D2 (Table 2). The relative abundances of these AOB groups were significantly higher in the latter phases (i.e., Phases E and D2) than in earlier phases (i.e., Phases B and C) (t-test, *p* < 0.001). Both *Nitrosomonas* and *Nitrosospira* targeted by Nso190 also increased from 8.1 ± 5.2% in Phase B to 26.4 ± 8.2% in Phase E and 28.8 ± 8.7% in Phase D2 (Table 2).

**Table 2:** Relative abundances of the different ammonia-oxidizing bacteria and nitrite-oxidizing bacteria obtained using HOPE

Method used		Phase A	Phase B	Phase C	Phase D1	Phase E	Phase D2	Spearman correlation between amplicon sequencing and HOPE data from primer with nearest coverage match
		Average relative abundance against the total bacteria ± standard deviation (%)						
	Ammonia-oxidizing bacteria (AOB)							
HOPE	NmoCL6a	6.7 ± 3.1	3.4 ± 1.3	2.9 ± 0.9	4.2 ± 2.6	23.2 ± 15.6	26.5 ± 9.5	
	Nmo218	3.7 ± 1.1	3.33 ± 2.6	Not detected	Not detected	0.01 ± 0.03	Not detected	
	Nse1472	1.6 ± 1.1	2.2 ± 1.8	0.3 ± 0.2	1.5 ± 1.5	18.9 ± 10.3	26.4 ± 5.0	
Amplicon sequencing	*Nitrosomonas*	2.0 ± 0.01	1.4 ± 1.1	0.1 ± 0.1	0.1 ± 0.2	6.8 ± 4.4	11.6 ± 3.0	*Nitrosomonas* with Nse1472: ρ = 0.91, *p* = 2.0 × 10^−14^
HOPE	Nsv443	Not detected	2.1 ± 1.8	4.3 ± 2.9	4.6 ± 3.0	3.3 ± 1.5	5.2 ± 1.5	
	Nso190	8.9 ± 1.4	8.1 ± 5.2	9.0 ± 4.8	11.5 ± 12.3	26.4 ± 8.2	28.8 ± 8.7	
Amplicon sequencing	*Nitrosospira*	0.03 ± 0.04	1.3 ± 1.1	2.8 ± 3.6	0.2 ± 0.8	0.5 ± 0.2	1.4 ± 0.6	*Nitrosospira* with Nsv443: ρ = 0.62, *p* = 5.9 × 10^−5^ *Nitrosomonas* and *Nitrosospira* with Nso190: ρ = 0.82, *p* = 6.7 × 10^−10^
	Nitrite-oxidizing bacteria (NOB)							
HOPE	Ntspa572	3.9 ± 0.3	4.3 ± 1.2	1.2 ± 1.6	Not detected	Not detected	0.01 ± 0.03	
	Ntspa712	6.3 ± 2.3	9.6 ± 3.1	3.7 ± 4.6	0.9 ± 1.8	2.3 ± 2.9	2.7 ± 1.9	
Amplicon sequencing	*Nitrospira*	10.5 ± 1.1	10.3 ± 2.7	2.2 ± 2.5	0.03 ± 0.02	0.003 ± 0.005	0.01 ± 0.02	*Nitrospira* with Ntspa712: ρ = 0.56, *p* = 3.4 × 10^−4^ *Nitrospira* with Ntspa572: ρ = 0.86, *p* = 3.1 × 10^−11^
HOPE	Nit1017	8.8 ± 3.5	4.8 ± 3.1	4.6 ± 0.8	4.7 ± 3.4	9.0 ± 3.9	8.4 ± 3.6	
	Nit1000	Not detected	1.0 ± 1.3	1.1 ± 0.3	0.5 ± 0.3	0.1 ± 0.3	Not detected	
Amplicon sequencing	*Nitrobacter*	Not detected	0.022 ± 0.05	0.01 ± 0.01	0.002 ± 0.002	Not detected	Not detected	*Nitrobacter* with Nit1017: ρ = −0.40, *p* = 0.02 *Nitrobacter* with Nit1000: ρ = 0.68, *p* = 6.1 × 10^−6^

The relative abundance of selected targets further correlated with the amplicon sequencing data

However, the increase in the relative abundance of Nso190-targets is mainly due to the increase in the relative abundance of *Nitrosomonas*. This is because *Nitrosospira* detected by primer Nsv443 remained stable in its relative abundance against the total community throughout the operational phases (Phase A: not detected; Phase B: 2.1 ± 1.8%; Phase C: 4.3 ± 2.9%; Phase D1: 4.6 ± 3.0%; Phase E: 3.3 ± 1.5%; Phase D2: 5.2 ± 1.5%). The changes in the relative abundance of Nsv443-targeted *Nitrosospira* were not significantly different across Phases B to D2 (t-test, *p* > 0.1).

### Abundance of NOB and *Nitrospira*-affiliated comammox in biofilm reactor quantified by HOPE throughout the operational phases

Vector analysis performed on the same nMDS plot also showed that samples collected from the early operational phases A and B were higher in relative abundance of NOB targeted by either Ntspa712 or Ntspa572. Both primers targeted *Nitrospirae* at the same hierarchical level but with different coverage (Additional file 1). Both primers also target at least one of the three recently identified comammox bacteria (i.e., Candidatus *N. inopinata*). The average relative abundance of *Nitrospira* targeted by Ntspa572 was 3.9 ± 0.3% in Phase A and 4.3 ± 1.2% in Phase B, and was significantly higher (*p* < 0.05) than that detected in Phases C (1.2 ± 1.6%), D1 and E (not detected), and D2 (0.01 ± 0.03%). Similarly, the relative abundance of *Nitrospira* targeted by Ntspa712 was 6.3 ± 2.3% in Phase A and 9.6 ± 3.1% in Phase B, and was significantly higher (*p* < 0.05) than that detected in Phases C (3.7 ± 4.6%), D1 (0.9 ± 1.8%), E (2.3 ± 2.9%) and D2 (2.7 ± 1.9%).

In contrast to *Nitrospira*, *Nitrobacter* detected by the Nit1017 primer increased in its relative abundance from 4.8 ± 3.2% in Phase B to 8.4 ± 3.6% in Phase D2 (Table 2). However, the Nit1000 primer did not perform in a similar manner as the Nit1017 primer despite both primers having similar detection coverage of most *Nitrobacter* spp. (Table 1). The Nit1000 primer consistently failed to detect any *Nitrobacter* in the latter operational phases, and in samples that were detected with *Nitrobacter*, the relative abundance was more than 2-fold lower than that detected by the Nit1017 primer.

### Validation of HOPE dataset obtained for trickling biofilm reactor with 16S rRNA gene-based amplicon sequencing

Based on the HOPE primer coverage shown in Additional file 1, the relative abundance of genera *Nitrosomonas*, *Nitrosospira, Nitrobacter* and *Nitrospira* obtained by 16S rRNA gene-based amplicon sequencing was respectively compared against the relative abundance detected by Nse1472 (*Nitrosomonas*), Nsv443 (*Nitrosospira*), Nit1000 or Nit1017 (*Nitrobacter*), and Ntsp572 or Ntspa712 (*Nitrospira*) HOPE primers. The relative abundance of *Nitrosomonas* and *Nitrospira* obtained by 16S rRNA gene-based amplicon sequencing was also compared against that detected by Nso190 HOPE primer.

Generally, the relative abundance obtained by HOPE differed from that detected by amplicon sequencing but both datasets exhibited good Spearman’s rank correlation (Table 2). To illustrate, the average relative abundance of *Nitrosomonas* reported by 16S rRNA gene-based amplicon sequencing was 2.0%, 1.4%, 0.1%, 0.1%, 6.8% and 11.6% in Phase A, B, C, D1, E and D2, respectively (Table 2). The average relative abundance of *Nitrosomonas* targeted by HOPE primer Nse1472 was 1.6%, 2.2%, 0.3%, 1.5%, 18.9% and 26.4% in Phase A, B, C, D1, E and D2, respectively. Similarly, the summation of relative abundance of *Nitrosomonas* and *Nitrosospira* obtained by amplicon sequencing differed from that detected by Nso190 HOPE primer (Table 2), although the datasets showed a Spearman’s rank correlation coefficient of ρ = 0.82.

For NOB, although Nit1017 detected higher relative abundance of *Nitrobacter* than Nit1000 HOPE primer, the results obtained from Nit1017 did not correlate positively with those detected by amplicon sequencing (ρ = −0.40). Instead, *Nitrobacter* spp. were detected in a very low abundance of <0.1% throughout the operational phases by amplicon sequencing and coincides with the range of relative abundance detected by HOPE using Nit1000 primer (ρ = 0.68). In addition, the average relative abundance of *Nitrospira* reported by amplicon sequencing decreased from 10.5% to 0.01% from Phase A to D2, and the same decrease in relative abundance targeted by Ntspa572 was observed, albeit with different relative abundance that decreased from 3.9% to 0.01%.

### Changes in biofilm reactor microbial community characterized by 16S rRNA gene-based amplicon sequencing

The total microbial communities of the trickling biofilm reactor were characterized by 16S rRNA gene-based amplicon sequencing (Fig. 2b). The nMDS plot generated by this data was similar to that which was obtained by HOPE in that the multivariate clusters were differentiated according to the operational phases of the reactor (Fig. 2b). Additional information was also obtained from the amplicon sequencing analysis. To illustrate, *Nitrososphaera*, which represent ammonia-oxidizing archaea, experienced a significant decrease in the relative abundance from 0.1% during early operational phases (Fig. 2b) to negligible levels during the late operational phases (*p* = 0.004). Other microbial populations that significantly decreased in their relative abundances across the different operational phases included unclassified Actinobacteria, Clostridiaceae I, Rhodospirillales and Deltaproteobacteria (t-test, *p* < 0.005). Unclassified Xanthomonadaceae had the highest relative abundance of 15.0 ± 5.9% during Phases C and D1, and averaged a relative abundance of 4.6 ± 3.1% in the remaining phases. Unclassified Burkholderiales increased significantly from 2.0 ± 1.2% in Phases A through D1 to 23.0 ± 10.8% in Phases D2 and E (*p* = 5.4 × 10^−6^).

### Performance of trickling nitrification biofilm reactor

**Fig. 3:**
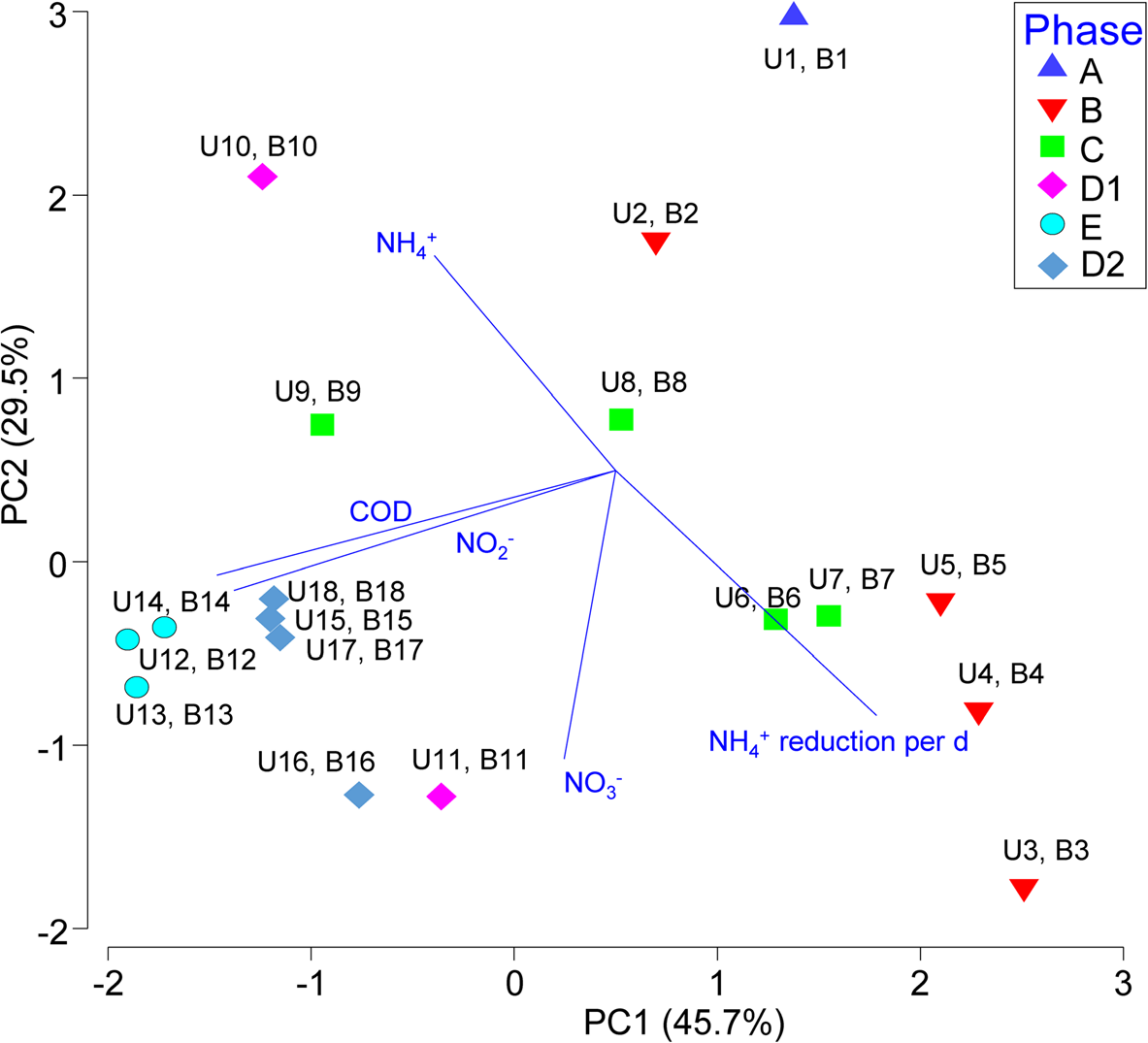
Principal component analysis (PCA) of the measured water quality parameters in the effluent stream throughout the operational phases. The PCA plot suggests that there was high ammonium NH_4_^+^ oxidation during Phases B and C but an accumulation of nitrite and an increase in the chemical oxygen demand (COD) during the late operational phases D2 and E

**Fig. 4:**
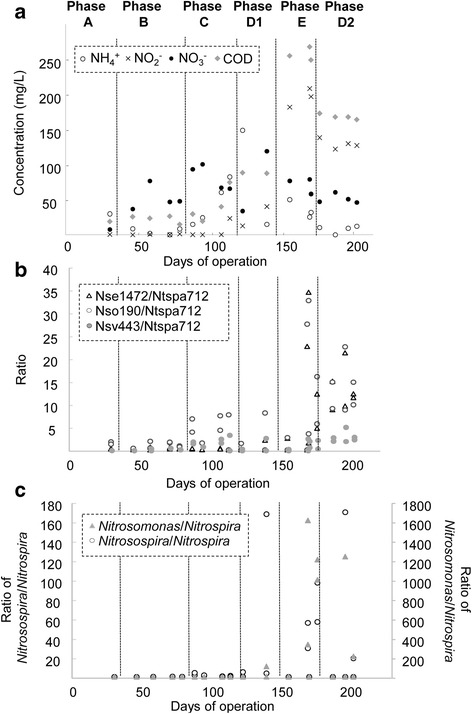
Correlative trends between effluent quality of the trickling nitrification biofilm reactor and the abundance ratio of predominant AOB and NOB. Concentrations of ammonium (NH_4_^+^), nitrite (NO_2_^−^), nitrate (NO_3_^−^) and chemical oxygen demand (COD) in the effluent stream of the trickling nitrification biofilm reactor column throughout the different operational phases (**a**). The ratio of Nse1472-, Nso190- and Nsv443-targeted AOB against Ntspa712-targeted NOB, obtained using HOPE (**b**). The ratio of *Nitrosospira* and *Nitrosomonas* against *Nitrospira*, obtained using amplicon sequencing (**c**)

Based on the collated water quality data, effluent from the trickling nitrification biofilm reactor clustered into the six operational phases, which corresponded with the influent ammonium content (Fig. 3). As the influent ammonium content was increased stepwise from 40 mg/L to 200 mg/L, the COD in the effluent also increased and was significantly higher in the effluent collected during Phase D1, D2 and E compared to those collected during Phase A and C (t-test, *p* = 2.1 × 10^−7^, Fig. 4a). Ammonium removal was optimally achieved at an average of 44% reduction per day during days 59 through 109 of operation (Phases B and C), which corresponded with an influent concentration of 80–110 mg/L (i.e., U3-U8). During these phases, ammonium was primarily converted to nitrate (average concentration 71.9 ± 21.5 mg/L) with minimal nitrite accumulation in the effluent (0.41 ± 0.45 mg/L). Subsequently, the trickling biofilm reactor experienced deterioration in its nitrification efficacy as influent ammonium content increased to 150 mg/L and 200 mg/L (Phases D and E). Ammonium was only partially converted to nitrate and an accumulation of nitrite was observed in the effluent collected during these phases (i.e., U11-U18). Nitrite levels in the effluent during the two latter phases were significantly higher than during Phases A through C (t-test, *p* = 9.9 × 10^−9^, Fig. 4a).

### Correlative trends of AOB and NOB proportions with reactor performance

The ratios in the relative abundance of these AOB (i.e., Nse1472, Nsv443 and Nso190) and NOB targeted by Ntspa712 HOPE primer were further evaluated and compared to the reactor performance based on its water quality parameters (Fig. 4a and b). It was determined that the average ratio of AOB targeted by Nse1472 and Nso190 normalized against Ntspa712-targeted NOB was 0.2 and 2.5, respectively, during the phases that corresponded to stable reactor performance as evidenced by low nitrite content in the effluent (i.e., Phases A through C). During Phases E and D2, which corresponded with deterioration in the reactor performance, the ratio of AOB targeted by Nse1472 and Nso190 normalized against Ntspa712-targeted NOB increased to 13.0 and 14.0, respectively. There was, however, a less significant increase in the ratio of AOB targeted by Nsv443 against Nit1017-targeted NOB (Fig. 4b). These ratios obtained using HOPE were compared against those generated from 16S rRNA-based amplicon sequencing data (Fig. 4c). The ratio of the relative abundance of *Nitrosomonas* compared against *Nitrospira* (including *Nitrospira*-affiliated comammox) as determined by amplicon sequencing was 0.12 during the stable operational phases. This ratio markedly increased to 948 when the reactor deteriorated in performance. Likewise, the ratio of *Nitrosospira* normalized against *Nitrospira* was observed to be 0.66 during Phase A to C, and increased to 72.7 in the latter phases.

### Detection of AOB and NOB in WWTP samples using HOPE and amplicon sequencing

Both HOPE and amplicon sequencing revealed that the activated sludge from the local full-scale WWTP had lower relative abundances of *Nitrosomonas*, *Nitrosospira* and *Nitrobacter* compared to Ntspa712-targeted *Nitrospira* (Table 3). Nitrate concentrations were consequentially higher in the effluent (*p* < 0.0001) than in the influent and chlorinated effluent. HOPE and amplicon sequencing were further used to evaluate the nitrifying bacterial populations present in these wastewater samples. Similar to the activated sludge, the wastewaters did not have any detectable *Nitrobacter* but instead, had relatively higher abundances of *Nitrospira* compared to *Nitrosomonas* and *Nitrosospira*. To illustrate, the relative abundance of Ntspa712-targeted NOB ranged from 3.1% in the influent to 1.0% in the chlorinated effluent. This relative abundance in *Nitrospira* was at least 2 times higher than the AOB targeted by Nse1472 and Nsv443 (Table 3). Amplicon sequencing also showed a relatively higher abundances of *Nitrospira* compared to both AOB (i.e., *Nitrosomonas* and *Nitrosospira*) in the three types of wastewater streams.

**Table 3:** Water quality for the influent, effluent and chlorinated effluent samples collected from the wastewater treatment plant and the corresponding relative abundances of AOB and NOB as quantified using the HOPE and amplicon sequencing approach

	Total nitrogen, TN (mg/L)	NH_4_^+^-N (mg/L)	NO_3_^-^-N (mg/L)	Nse1472	Nsv443	Ntspa712	Ntspa572	Nit1000	*Nitrosomonas*	*Nitrosospira*	*Nitrobacter*	*Nitrospira*
				Relative abundance (%) with respect to Eub338Ia (HOPE data)					Relative abundance (%) with respect to total bacteria (Amplicon sequencing data)			
Influent	17 ± 1.3	12.3 ± 2.4	2.9 ± 0.8	0.28 ± 0.43	Not detected	3.1 ± 1.0	0.6 ± 0.5	Not detected	Not detected	Not detected	Not detected	0.02 ± 0.04
Effluent	17 ± 1.4	Not applicable	14 ± 2.3	0.61 ± 0.73	1.1 ± 2.9	2.3 ± 1.7	2.0 ± 1.6	Not detected	0.004 ± 0.007	0.008 ± 0.014	Not detected	1.8 ± 2.6
Chlorinated effluent	14 ± 2.4		8.6 ± 0.9	0.03 ± 0.07	0.2 ± 0.5	1.0 ± 1.3	1.6 ± 2.2	Not detected	0.01 ± 0.02	0.003 ± 0.008	Not detected	1.0 ± 1.5
Oxic sludge	Not applicable			0.23 ± 0.13	0.21 ± 0.28	8.1 ± 3.4	4.8 ± 2.4	0.17 ± 0.21	Not detected	Not detected	Not detected	6.4 ± 1.7

## Discussion

This study demonstrates the development of HOPE as a high-throughput method to quantify for the nitrifying bacterial populations including AOB, NOB and *Nitrospira*-affiliated comammox. Along with the AOA, which was not targeted by the HOPE approach in this study, these nitrifying populations play an important role in the activated sludge process to convert ammonia in the untreated wastewater to nitrite and nitrate. The nitrate is subsequently converted to dinitrogen and nitrogen by denitrifiers. Collectively, the nitrifying and denitrifying populations reduce the total nitrogenous content in wastewater to a level permissible for discharge or reuse. It has previously been hypothesized that the theoretical AOB/NOB ratio in a nitrification process would be 2 due to the differences in electron generation and biomass yield between AOB and NOB [22–25]. Simultaneous nitrification/denitrification can possibly increase this ratio to 3 (i.e., more NOB than AOB) [26]. However, the inverse may have a potentially detrimental impact on overall functionality of the nitrification system, especially since excess AOB can lead to accumulation of the hydroxylamine intermediate which is inhibitory towards NOB [3]. As such, the HOPE method is developed with the intention to serve as a practically applicable tool for monitoring the AOB and NOB ratio and hence used to infer the efficiency of the nitrification process in wastewater treatment systems.

For this purpose, the HOPE primers were designed to target *Nitrosomonas*, *Nitrosospira*, *Nitrobacter* and *Nitrospira* at the order, subcluster and/or genus level, and the relative abundance at each hierarchical taxonomical level was normalized against that of the total bacteria. Primers were arranged into multiple reaction tubes that involve multiplexing of up to four targets per reaction, and tested against samples collected from a trickling nitrification biofilm reactor. It was observed from the HOPE data that the relative abundance of *Nitrosomonas* increased with increasing NH_4_^+^ concentrations while abundances of *Nitrosospira* did not. In contrast to the AOB, the NOB genus *Nitrospira* was detrimentally affected by the shock loading of the reactor. During the stable phases B and C, *Nitrospira* targeted by the Ntspa572-primer was more predominant than *Nitrobacter*. However, with the accumulation of nitrite in the late operational phases, *Nitrospira* was depleted while *Nitrobacter*, targeted by the Nit1000-primer, remained relatively stable in its relative abundance against the total bacteria. This observation coincides with results reported by Knapp and Graham [27], in which *Nitrospira* was observed to be more perturbed than *Nitrobacter* during a destabilizing experiment [27]. In addition, given that both Ntspa572 and Ntspa712 HOPE primers target at least one of the three recently identified comammox (Table 1), the higher relative abundance of HOPE-targeted *Nitrospira* than *Nitrobacter* is likely to suggest that comammox is contributing to an equally important nitrification role in this reactor. Similarly, the depletion in the collective relative abundance of Ntspa572-targeted *Nitrospira* suggests the susceptibility of comammox to shock loading events.

The high NH_4_^+^ content exposure appeared to affect specific groups of AOB and NOB differently. It is generally thought that *Nitrospira*-like bacteria are k-strategists with a high affinity for NO_2_^−^ and oxygen, and reach high densities under substrate-limited conditions. This is in contrast to the *Nitrobacter* species, which are r-strategists with a lower NO_2_^−^ and oxygen affinity and outcompete *Nitrospira* only at higher substrate concentrations [28–30]. An additional postulation to account for the better survival of *Nitrobacter* during shock loading events is its greater metabolic diversity under stressed conditions [27, 31]. This genus exhibits versatile metabolism and is able to grow either mixotrophically or chemoorganotrophically [32]. Although *Nitrobacter* was able to withstand shock loading and increase in its relative abundance as quantified by HOPE, it was observed that the increase in its relative abundance did not correlate with any decrease in nitrite content of the effluent. Furthermore, the increase in *Nitrobacter* did not correlate with a subsequent conversion of nitrite to nitrate in the effluent. This suggests that *Nitrobacter* may not be playing as important of a role as *Nitrospira* and comammox in nitrite oxidation in the wastewater treatment process.

Given that both *Nitrosomonas* and *Nitrospira* seem to correlate with nitrification functionality, the HOPE-obtained relative abundances of both groups were further expressed as a proportional ratio. The ratio of *Nitrosomonas* targeted by either Nse1472 or Nso190 HOPE primer against Ntspa572-targeted *Nitrospira* ranged from 0.2 to 2.5 in the nitrification biofilm reactor during the phases that corresponded to stable reactor performance as evidenced by the low nitrite content in the effluent. However, the exceedingly high ammonium content of the later phases of operation (i.e., Phase E and D2) resulted in a toxic shock response by the nitrifying bacterial populations, which correlated with an observed increase in the AOB/NOB ratio. This ratio increased to a range of 13.0 to 13.7 when the bioreactor performance deteriorated. Based on energetic calculations, it was proposed that a theoretical AOB/NOB ratio of 2 should be obtained during a stable and functional nitrification process (i.e., Phase A and B) [26]. This theoretical ratio was in agreement with the observation made in this study, and reiterates the need to maintain an optimal proportion of AOB to NOB in a well-performing nitrification process.

The HOPE method had been previously validated against other conventional methods such as qPCR and FISH [7, 8, 11]. In this study, to validate the data obtained from HOPE, the relative abundances of nitrifying bacterial populations in the trickling biofilter and in samples collected from a full-scale WWTP were further validated against results from amplicon sequencing (Tables 2 and 3). The observed difference in the abundance values between HOPE and amplicon sequencing is likely due to differences in the primer coverage for the individual AOB and NOB groups (Additional file 1). For example, Nso190 targets 27.2% of the *Nitrosomonas* and 17.4% of the *Nitrosospira* sequences collated in the RDP Classifier database. In contrast, the universal primers 515F and 907R used for amplicon sequencing target a higher percentage of *Nitrosomonas* and *Nitrosospira*. Furthermore, the relative abundances reported by HOPE were expressed by normalizing against total bacteria as detected by universal primers that differed from those used in amplicon sequencing. Although the relative abundances determined by both methods varied in terms of the absolute values, both datasets showed good Spearman’s rank correlation. Furthermore, multivariate nMDS plots from both HOPE and amplicon sequencing also showed good correlation at a significant confidence level (ρ = 0.79, *p* = 0.001). This indicates that the observed trends in the nitrifying bacterial population dynamics revealed by both methods were well aligned.

Although similar conclusions were obtained from both HOPE and amplicon sequencing with regards to the nitrifying population dynamics in the presence of shock loading, a limitation of the HOPE approach is that it can only detect bacterial targeted by the designed primers. For instance, through the use of amplicon sequencing and not HOPE, it was determined that the detrimental effect of the shock loading event on *Nitrospira* can possibly be due to its competition for substrates (e.g. oxygen) with heterotrophic bacteria. Such heterotrophic bacteria included unclassified Burkholderiales and unclassified Xanthomonadaceae, which increased in relative abundance throughout all operational phase. Furthermore, the HOPE technique developed in this study does not contain primers that target the AOA. Although AOA had been previously found abundant in wastewater treatment systems [33, 34], both genus *Nitrososphaera* and *Nitrosopumilus* were found to be in low abundance (< 0.1%) in the samples when evaluated by high-throughput amplicon sequencing. Further optimization on the HOPE technique would be required to design a range of AOA-targeting primers, as well as to improve its current detection limits of 0.1% so as to facilitate the quantification of potentially low abundance species.

Regardless, an advantage of the HOPE approach is that new primers, for example those designed to target the different sublineages of *Nitrospira* and *Nitrosomonas* that were selected for by varying nitrite and ammonium concentrations [35–37], can be easily added to the multiplexing tube arrangement when these primers become available. The additional depth offered by high-throughput amplicon sequencing would be useful as a tool for periodically benchmarking HOPE-based results obtained on a more frequent basis. Although this study did not attempt to challenge the limits of high-throughput multiplexing capability of HOPE, the method can in theory perform up to 32-plexes per reaction tube, and modifications to the HOPE approach can be efficiently made to facilitate the simultaneous detection of the expanding list of nitrifying bacteria. Furthermore, this study demonstrated a good correlation between the results obtained from the HOPE approach and high-throughput sequencing. Thus, depending on the intended purpose of an experimental or practical study, HOPE may be particularly useful for the frequent tracking of the occurrence and abundance of selected microbial populations without the need to perform high-throughput amplicon sequencing. This in turn reduces the need to sieve through a full range of microbial population data for every sampling event.

## Conclusion

In summary, this study demonstrates the applicability and adaptability of HOPE for assessing abundances of predominant AOB and NOB groups that have been identified as integral to wastewater treatment systems. This method allows for the simultaneous monitoring of relative abundances of AOB, NOB and comammox groups, which can be used to provide indicative data of nitrification performance and efficiency. Coupled with previous application of HOPE to a variety of target bacteria and sample types [7–11], this study demonstrates the versatility and applicability of HOPE for a wide range of microbial ecology studies.

## Additional file

Additional file 1: Target coverage for the different primers used in this study. (PDF 1668 kb)

## Abbreviations

AOA: Ammonia-oxidizing archaea
AOB: Ammonia-oxidizing bacteria
COD: Chemical oxygen demand
ddNTP: Dideoxynucleoside triphosphate
dPCR: Digital polymerase chain reaction
FISH: Fluorescent in-situ hybridization
HOPE: Hierarchical oligonucleotide primer extension
MBR: Membrane bioreactor
NH_4_^+^-N: Ammonium
nMDS: Non-metric threshold multidimensional scaling
NO_2_^-^-N: Nitrite
NO_3_^-^-N: Nitrate
NOB: Nitrite-oxidizing bacteria
nt: Nucleotides
PBS: Phosphate buffer saline
PCA: Principal component analysis
qPCR: Quantitative polymerase chain reaction
RDP: Ribosomal database project
rSAP: Recombinant shrimp alkaline phosphatase
TN: Total nitrogen
WWTP: Wastewater treatment plant
