# Supplementary material for: Application of hierarchical oligonucleotide primer extension (HOPE) to assess relative abundances of ammonia- and nitrite-oxidizing bacteria
Source: BMC Microbiol. 2017 Apr 4;17:85. doi: 10.1186/s12866-017-0998-2 (PMC5381152; doi:10.1186/s12866-017-0998-2)
Supplement: Additional file 1: — Target coverage for the different primers used in this study. (PDF 1668 kb) [file 12866_2017_998_MOESM1_ESM.pdf]

| Ntspa712 | 5’-CGCCTTCGCCACCGGCCTTCC -3’ |
|---|---|

Root (5089/3333501) ; Bacteria (5089/3070243) ; Nitrospirae (4451/8171) ; "Nitrospira" (4451/8171) ; "Nitrospirales" (4451/8171) ; "Nitrospiraceae" (4451/8171)

**Hierarchy View:**

▼ **genus** Nitrospira (2358/5378) (hits/total searched) [list results for this node]

S000000821 Nitrospira sp.; AJ224042
S000001332 Nitrospira sp.; Y14640
S000004359 Nitrospira sp.; Y14642
S000005124 Nitrospira moscoviensis (T); X82558
S000006640 Nitrospira sp.; Y14636
S000007351 Nitrospira sp.; Y14641
S000008534 Nitrospira sp.; AJ224041
S000009675 Nitrospira sp.; Y14644
S000010052 Nitrospira sp.; Y14637
S000010421 Nitrospira sp.; AJ224038
S000010844 Nitrospira sp.; Y14638
S000012390 Nitrospira sp.; AJ224039
S000015738 Nitrospira sp.; Y14643
S000016078 Nitrospira sp.; AJ224040
S000021615 Nitrospira sp.; Y14639
S000024494 Nitrospira sp.; AJ224044
S000024499 Nitrospira sp.; AJ224043
S000024623 Nitrospira sp.; AJ224045
S000024748 Nitrospira sp.; AJ224046
S000025371 uncultured bacterium 1405-19; AJ007652
S000040958 rhizosphere soil bacterium RSC-II-52; AJ252684
S000100950 uncultured Nitrospira sp.; JG37-AG-131; AJ519405
S000102350 uncultured bacterium; JG37-AG-70; AJ519620
S000106404 uncultured Nitrospirae bacterium; JG36-TzT-13; AJ534638
S000130382 uncultured bacterium; C4-K18; AJ421207
S000131017 uncultured bacterium; C3-K5; AJ421201
S000144745 uncultured Nitrospira sp.; JG36-GS-2; AJ582041
S000249705 uncultured bacterium; EA1B1; AY186068
S000261709 uncultured soil bacterium; 605; AY493909
S000273128 uncultured bacterium; 1013-28-CG51; AY532586
S000273576 uncultured bacterium; 1013-28-CG50; AY532585
S000331342 uncultured bacterium; Kazan-1B-07/BC19-1B-07; AY592084
S000336675 uncultured bacterium A-11; AF033558
S000336676 uncultured bacterium A-4; AF033559
S000338023 uncultured soil bacterium S004; AF128670
S000338082 uncultured soil bacterium C059; AF128729
S000341118 uncultured Green Bay ferromanganous micronodule bacterium MNC2; AF293010
* S000341119 uncultured Green Bay ferromanganous micronodule bacterium MNB2; AF293011
* S000341120 uncultured Green Bay ferromanganous micronodule bacterium MNF8; AF293012
* S000341121 uncultured Green Bay ferromanganous micronodule bacterium MNA3; AF293013
S000341501 uncultured bacterium PHOS-HE34; AF314422
S000341784 unidentified bacterium wb1_D06; AF317756
S000341786 unidentified bacterium wb1_C02; AF317758
S000341792 unidentified bacterium wb1_F07; AF317764
S000345624 uncultured bacterium; G19; AF407702
* S000351038 uncultured bacterium; JCL9-22; AF499363
* S000351107 uncultured bacterium; BL-59; AF499432
S000351613 uncultured bacterium; C6-K31; AJ421160
S000357855 uncultured bacterium; DSSD16; AY328715
S000357883 uncultured bacterium; DSSD45; AY328743
S000357900 uncultured bacterium; DSSD62; AY328760
S000366366 uncultured Nitrospirae bacterium; DGGE gel band ESR BR 14; AF540049
S000366371 uncultured Nitrospirae bacterium; DGGE gel band ESR BR 19; AF540054
S000388268 Nitrospira cf. moscoviensis SBR1015; AF155152
S000388269 Nitrospira cf. moscoviensis SBR1024; AF155153
S000388270 Nitrospira cf. moscoviensis SBR2016; AF155154
S000388271 Nitrospira cf. moscoviensis SBR2046; AF155155
* S000397830 uncultured Nitrospirae bacterium; DGGE band RWA12; AY145869
S000397857 Nitrospirales bacterium K21; AY146671
S000398160 uncultured Nitrospira sp.; WYO26C; AY150907
S000425892 uncultured Nitrospira sp.; AY741094
S000427926 Nitrospira sp.; AF035813
S000471701 uncultured bacterium; D023010; AJ617918
S000471702 uncultured bacterium; D14423; AJ617919
S000471703 uncultured bacterium; D023011; AJ617920
S000476573 uncultured Nitrospira sp.; BDC1_F10; AY689544
S000476652 uncultured Nitrospira sp.; BDC3SG06; AY689623

| Ntspa572 | 5’-AACCGCCTACGCTCCCTG -3’ |
|---|---|

**Lineage** *(click node to return it to hierarchy view)*:

Root (1849/3333501) ; Bacteria (1849/3070243) ; Nitrospirae (1835/8171) ; "Nitrospira" (1835/8171) ; "Nitrospirales" (1835/8171) ; "Nitrospiraceae" (1835/8171)

**Hierarchy View:**

▼ **genus** Nitrospira (1835/5378) (hits/total searched) [list results for this node]

S000000821 Nitrospira sp.; AJ224042
S000005124 Nitrospira moscoviensis (T); X82558
S000009675 Nitrospira sp.; Y14644
S000012390 Nitrospira sp.; AJ224039
S000024494 Nitrospira sp.; AJ224044
S000025371 uncultured bacterium 1405-19; AJ007652
S000025716 uncultured bacterium 1404-16; AJ007648
S000040958 rhizosphere soil bacterium RSC-II-52; AJ252684
S000106404 uncultured Nitrospirae bacterium; JG36-TzT-13; AJ534638
S000131017 uncultured bacterium; C3-K5; AJ421201
S000140627 uncultured soil bacterium; 1164-1; AY326515
S000140948 uncultured soil bacterium; 1163-1; AY326514
S000144745 uncultured Nitrospira sp.; JG36-GS-2; AJ582041
S000251489 unidentified bacterium; Neu4P3-38; AJ518433
S000252064 unidentified bacterium; Neu4P1-67; AJ518386
S000252632 unidentified bacterium; Mul2P1-48; AJ518201
S000261709 uncultured soil bacterium; 605; AY493909
S000262278 uncultured soil bacterium; 1183; AY493911
S000262850 uncultured soil bacterium; 339; AY493910
S000273576 uncultured bacterium; 1013-28-CG50; AY532585
S000331342 uncultured bacterium; Kazan-1B-07/BC19-1B-07; AY592084
S000338023 uncultured soil bacterium S004; AF128670
S000341118 uncultured Green Bay ferromanganous micronodule bacterium MNC2; AF293010
* S000341120 uncultured Green Bay ferromanganous micronodule bacterium MNF8; AF293012
* S000341121 uncultured Green Bay ferromanganous micronodule bacterium MNA3; AF293013
S000341786 unidentified bacterium wb1_C02; AF317758
S000342310 uncultured bacterium; LCo23; AF337199
S000342509 uncultured Nitrospira sp.; 4-11; AF351228
* S000351038 uncultured bacterium; JCL9-22; AF499363
* S000351107 uncultured bacterium; BL-59; AF499432
S000351613 uncultured bacterium; C6-K31; AJ421160
S000353360 uncultured soil bacterium; S085; AY037608
S000353368 uncultured soil bacterium; S096; AY037616
S000357855 uncultured bacterium; DSSD16; AY328715
S000357883 uncultured bacterium; DSSD45; AY328743
S000357900 uncultured bacterium; DSSD62; AY328760
S000366366 uncultured Nitrospirae bacterium; DGGE gel band ESR BR 14; AF540049
S000366371 uncultured Nitrospirae bacterium; DGGE gel band ESR BR 19; AF540054
S000388268 Nitrospira cf. moscoviensis SBR1015; AF155152
S000388269 Nitrospira cf. moscoviensis SBR1024; AF155153
S000388271 Nitrospira cf. moscoviensis SBR2046; AF155155
S000402935 uncultured bacterium; CCU23; AY221079
S000471700 uncultured bacterium; D02307; AJ617917
S000471701 uncultured bacterium; D023010; AJ617918
S000471702 uncultured bacterium; D14423; AJ617919
S000471703 uncultured bacterium; D023011; AJ617920
S000492977 uncultured Nitrospirae bacterium; AKYG586; AY921743
S000492978 uncultured Nitrospirae bacterium; AKYG1809; AY921744
* S000514101 uncultured Arthrobacter sp.; 24; AY942952
S000514160 uncultured bacterium; 37; AY943011
* S000514190 uncultured Arthrobacter sp.; 24; AY943041
S000535842 uncultured Nitrospira sp.; Nsp2; AY876618
S000535843 uncultured Nitrospira sp.; Nsp9; AY876619
S000535844 uncultured Nitrospira sp.; Nsp11; AY876620
S000535845 uncultured Nitrospira sp.; Nsp1; AY876621
S000535846 uncultured Nitrospira sp.; Nsp27; AY876622

| Ntspa1429 | 5’-TGGCTTGGGCGACTTCAG -3’ |
|---|---|

**Lineage** *(click node to return it to hierarchy view)*:

Root (119/3333501) ; Bacteria (119/3070243) ; Nitrospirae (107/8171) ; "Nitrospira" (107/8171) ; "Nitrospirales" (107/8171) ; "Nitrospiraceae" (107/8171)

**Hierarchy View:**

▼ genus Nitrospira (107/5378) (hits/total searched) [list results for this node]

S000001332 Nitrospira sp.; Y14640
S000004359 Nitrospira sp.; Y14642
S000006640 Nitrospira sp.; Y14636
S000007351 Nitrospira sp.; Y14641
S000008534 Nitrospira sp.; AJ224041
S000010052 Nitrospira sp.; Y14637
S000010421 Nitrospira sp.; AJ224038
S000010844 Nitrospira sp.; Y14638
S000015738 Nitrospira sp.; Y14643
S000021615 Nitrospira sp.; Y14639
S000024499 Nitrospira sp.; AJ224043
S000024623 Nitrospira sp.; AJ224045
S000024748 Nitrospira sp.; AJ224046
S000336675 uncultured bacterium A-11; AF033558
S000336676 uncultured bacterium A-4; AF033559
S000341501 uncultured bacterium PHOS-HE34; AF314422
S000348943 uncultured bacterium; PO8; AF440288
S000388270 Nitrospira cf. moscoviensis SBR2016; AF155154
S000425892 uncultured Nitrospira sp.; AY741094
S000537353 Candidatus Nitrospira defluvii; DQ059545
S000607302 uncultured bacterium; P3-N36; AB239557
S000607308 uncultured bacterium; Bu-N20; AB239563
S000607310 uncultured bacterium; Bu-N36; AB239565
S000656862 uncultured bacterium; AP19; AY921494
S000662948 uncultured bacterium; 71; DQ413129
S000668031 uncultured Nitrospira sp.; C4; DQ458783
S000686334 uncultured bacterium; C4; DQ499976
S000702219 uncultured Nitrospirae bacterium; 73; AB252941
S000702220 uncultured Nitrospirae bacterium; 369; AB252942
S000702221 uncultured Nitrospirae bacterium; 330; AB252943
S000702222 uncultured Nitrospirae bacterium; 406; AB252944
S000720826 uncultured Nitrospira sp.; BF9; DQ839324
S000749462 uncultured bacterium; ISS-3; EF095013
S000862151 uncultured Nitrospirae bacterium; Skagenf5; DQ640657
S001030603 Candidatus Nitrospira defluvii; Contig5882; EU559167
S001158011 uncultured bacterium; N1903_93; EU104347
S001224556 uncultured bacterium; FHINOA38/1-7682; FJ430192
S001294794 uncultured bacterium; TSBAR001_C14; AB486070
* S001318824 uncultured bacterium; B16; FJ660506
S001318828 uncultured bacterium; B21; FJ660510
S001318830 uncultured bacterium; B23; FJ660512
* S001318832 uncultured bacterium; B25; FJ660514
S001318833 uncultured bacterium; B26; FJ660515
* S001318921 uncultured bacterium; A208; FJ660604
S001353272 uncultured Nitrospira sp.; NSPI-2; AB500063
S001585993 uncultured bacterium; Kis08_048; GQ385296
S001614188 uncultured bacterium; K26C1-29; AB504576
S001614233 uncultured bacterium; K29J2-18; AB504621
S001614262 uncultured bacterium; K29C2-17; AB504650
S001614275 uncultured bacterium; K29G2-3; AB504663
S001614287 uncultured bacterium; K29G2-21; AB504675
* S001683724 uncultured bacterium; T1-102; GQ487925
* S001683728 uncultured bacterium; T1-126; GQ487929
S001683795 uncultured bacterium; V8-58; GQ487996
S001752975 uncultured bacterium; MFC_32E10; FJ624177
S001938902 uncultured bacterium; ambient_uncontrolled-12; GU454873
S001938972 uncultured bacterium; ambient_uncontrolled-82; GU454943
S002222769 Candidatus Nitrospira defluvii; FP929003
S002289961 Candidatus Nitrospira defluvii; FP929003
S002303062 uncultured bacterium; 147; HQ538637
S002337126 uncultured bacterium; F_SBR_72; HQ010847
S002373993 uncultured bacterium; T35; HM007548
S002437234 uncultured bacterium; Anxy8; HQ343213
S002495762 uncultured Nitrospirae bacterium; LJ-B96; JF319184
S002502514 uncultured bacterium; T1-28; JF502873
[illegible]

| Nit1017 | 5’-TGC TCC GAA GAG AAG GTC ACA -3’ |
|---|---|

**Lineage** *(click node to return it to hierarchy view)*:

Root (167/3333501) ; Bacteria (167/3070243) ; "Proteobacteria" (136/1031919) ; Alphaproteobacteria (136/254486) ; Rhizobiales (136/84204) ; Bradyrhizobiaceae (135/13197)

**Hierarchy View:**

▼ genus Nitrobacter (107/195) (hits/total searched) [list results for this node]

* S000265885 Nitrobacter sp. PBAB10; AY508475
* S000269985 Nitrobacter sp. PBAB17; AY508476
S000414380 Nitrobacter winogradskyi; W; L11661
S000414381 Nitrobacter sp.; LL; L11662
S000414382 Nitrobacter hamburgensis (T); X14; L11663
S000414627 Nitrobacter hamburgensis; Nb14; L35502
S000414631 Nitrobacter winogradskyi; L35506
S000414636 Nitrobacter sp.; R6; L35511
S000428396 Nitrobacter alkalicus (T); AN1; AF069956
S000428397 Nitrobacter alkalicus; AN2; AF069957
S000428398 Nitrobacter alkalicus; AN4; AF069958
S000428549 Nitrobacter sp. TH21; AF080257
* S000434952 Nitrobacter sp. KB215; AY055794
S000434953 Nitrobacter sp. PJN1; AY055795
S000434955 Nitrobacter sp. NRB5220; AY055797
S000487444 uncultured Rhizobiales bacterium; JAB SHC 117; AY694549
S000492976 uncultured alpha proteobacterium; AKYH732; AY921742
S000535828 uncultured Nitrobacter sp.; Nbac20; AY876604
S000535829 uncultured Nitrobacter sp.; Nbac20-3-12s; AY876605
S000535831 uncultured Nitrobacter sp.; Nbac20-4o14; AY876607
S000535832 uncultured Nitrobacter sp.; Nbac3-2; AY876608
S000535833 uncultured Nitrobacter sp.; Nbac3-2-4o12; AY876609
S000535841 uncultured Nitrobacter sp.; Nbac21; AY876617
S000568817 uncultured bacterium; UTFS_N_MP_RUN7_25; AB200974
S000608915 Nitrobacter vulgaris (T); type strain: DSM 10236; AM114522
S000770488 uncultured bacterium; PnDHS-18; AB290707
S000858444 Nitrobacter winogradskyi; Engel; AM286374
S000858446 Nitrobacter sp. Nato; AM286376
S000858447 Nitrobacter winogradskyi; F83KO; AM286377
S000858448 Nitrobacter vulgaris; 339; AM286378
S000858449 Nitrobacter sp. GT oman; AM286379
S000858450 Nitrobacter vulgaris; AB1; AM286380
S000858451 Nitrobacter vulgaris; K48; AM286381
S000858452 Nitrobacter vulgaris; K55; AM286382
S000858454 Nitrobacter sp. 5F/3; AM286384
S000858455 Nitrobacter sp. LIP; AM286385
S000858456 Nitrobacter sp. 4111/1; AM286386
S000858458 Nitrobacter sp. AB3; AM286388
S000858459 Nitrobacter winogradskyi; R1.30; AM286389
S000858460 Nitrobacter vulgaris; 329; AM286390
S000858461 Nitrobacter sp. Termite2; AM286391
S000858462 Nitrobacter sp. Yukatan; AM286392
S000858463 Nitrobacter vulgaris; C2; AM286393
S000858464 Nitrobacter sp. D6/13; AM286394
S000858465 Nitrobacter sp. lo acid; AM286395
S000858466 Nitrobacter hamburgensis; Y; AM286396
S000858467 Nitrobacter sp. 311; AM286397
S000858468 Nitrobacter vulgaris; BB3; AM286398
S000901726 Nitrobacter winogradskyi; Engel; AM292277
S000901728 Nitrobacter sp. Nato; AM292279

| Nit1000 | 5'-TGC GAC CGG TCA TGG -3' |
|---|---|

**Lineage** *(click node to return it to hierarchy view)*:

Root (153/3333501) ; Bacteria (153/3070243) ; "Proteobacteria" (153/1031919) ; Alphaproteobacteria (151/254486) ; Rhizobiales (151/84204) ; Bradyrhizobiaceae (148/13197)

**Hierarchy View:**

▼ **genus** Nitrobacter (117/195) (hits/total searched) [list results for this node]

* S000265885 Nitrobacter sp. PBAB10; AY508475
* S000269985 Nitrobacter sp. PBAB17; AY508476
* S000392359 Nitrobacter winogradskyi; IFO 14297; AF363288

S000397176 nitrite-oxidizing bacterium MPN2; AY135357
S000414380 Nitrobacter winogradskyi; W; L11661
S000414381 Nitrobacter sp.; LL; L11662
S000414382 Nitrobacter hamburgensis (T); X14; L11663
S000414627 Nitrobacter hamburgensis; Nb14; L35502
S000414631 Nitrobacter winogradskyi; L35506
S000414632 Nitrobacter winogradskyi; L35507
S000414636 Nitrobacter sp.; R6; L35511
S000428396 Nitrobacter alkalicus (T); AN1; AF069956
S000428397 Nitrobacter alkalicus; AN2; AF069957
S000428398 Nitrobacter alkalicus; AN4; AF069958
S000428549 Nitrobacter sp. TH21; AF080257
S000434953 Nitrobacter sp. PJN1; AY055795
S000434954 Nitrobacter winogradskyi; ATCC 14123; AY055796
S000434955 Nitrobacter sp. NRB5220; AY055797
S000487300 uncultured Nitrobacter sp.; 1; AY683484
S000487444 uncultured Rhizobiales bacterium; JAB SHC 117; AY694549
S000492976 uncultured alpha proteobacterium; AKYH732; AY921742
S000535828 uncultured Nitrobacter sp.; Nbac20; AY876604
S000535829 uncultured Nitrobacter sp.; Nbac20-3-12s; AY876605
S000535831 uncultured Nitrobacter sp.; Nbac20-4o14; AY876607
S000535832 uncultured Nitrobacter sp.; Nbac3-2; AY876608
S000535833 uncultured Nitrobacter sp.; Nbac3-2-4o12; AY876609
S000535841 uncultured Nitrobacter sp.; Nbac21; AY876617
S000568817 uncultured bacterium; UTFS_N_MP_RUN7_25; AB200974
S000608915 Nitrobacter vulgaris (T); type strain: DSM 10236; AM114522
S000770488 uncultured bacterium; PnDHS-18; AB290707
S000858444 Nitrobacter winogradskyi; Engel; AM286374
S000858446 Nitrobacter sp. Nato; AM286376
S000858447 Nitrobacter winogradskyi; F83KO; AM286377
S000858448 Nitrobacter vulgaris; 339; AM286378
S000858449 Nitrobacter sp. GT oman; AM286379
S000858450 Nitrobacter vulgaris; AB1; AM286380
S000858451 Nitrobacter vulgaris; K48; AM286381
S000858452 Nitrobacter vulgaris; K55; AM286382
S000858454 Nitrobacter sp. 5F/3; AM286384
S000858455 Nitrobacter sp. LIP; AM286385
S000858456 Nitrobacter sp. 4111/1; AM286386
S000858458 Nitrobacter sp. AB3; AM286388
S000858459 Nitrobacter winogradskyi; R1.30; AM286389
S000858460 Nitrobacter vulgaris; 329; AM286390
S000858461 Nitrobacter sp. Termite2; AM286391
S000858462 Nitrobacter sp. Yukatan; AM286392
S000858463 Nitrobacter vulgaris; C2; AM286393
S000858464 Nitrobacter sp. D6/13; AM286394
S000858465 Nitrobacter sp. lo acid; AM286395
S000858466 Nitrobacter hamburgensis; Y; AM286396

| NmoCL6a | 5'-AAGCATAAGGTCTTTCGATCCCCT -3' |
|---|---|

**Lineage** *(click node to return it to hierarchy view)*:

Root (437/3333501) ; Bacteria (437/3070243) ; "Proteobacteria" (432/1031919) ; Betaproteobacteria (430/217518) ; Nitrosomonadales (379/7530)

**Hierarchy View:**

**family** Nitrosomonadaceae (379/7522) (hits/total searched) [list results for this node]

- genus **Nitrosomonas** (49/911)
- genus **Nitrosospira** (37/5714)
- **unclassified_Nitrosomonadaceae** (293/897)

| NmoCL6b | 5’-GGATCAGGCTTGCGCCC -3’ |
| --- | --- |

**Lineage** *(click node to return it to hierarchy view)*:

Root (198344/3333501) ; Bacteria (198344/3070243) ; "Proteobacteria" (184478/1031919) ; Betaproteobacteria (67027/217518)

**Hierarchy View:**

- order Nitrosomonadales (1749/7530) (hits/total searched) [list results for this node]
  - family Nitrosomonadaceae (1749/7522)
    - ▶ genus Nitrosomonas (118/911)
    - ▶ genus Nitrosospira (1058/5714)
    - ▶ unclassified_Nitrosomonadaceae (573/897)
  - family Spirillaceae (0/7)
  - unclassified_Nitrosomonadales (0/1)

* NmoCL6b is not very specific and is likely to be targeting other non-AOB targets present in the wastewater ecosystem

## Non-specific targets

**Hierarchy View:**

- rootrank Root (198344/3333501) (hits/total searched) [list results for this node]
  - domain Bacteria (198344/3070243)
    - phylum "Actinobacteria" (154/349461)
    - phylum "Aquificae" (5/2085)
    - phylum "Bacteroidetes" (12050/388464)
    - phylum "Caldiserica" (0/361)
    - phylum "Chlamydiae" (0/1538)
    - phylum "Chlorobi" (0/1556)
    - phylum "Chloroflexi" (73/42562)
    - phylum "Chrysiogenetes" (0/13)
    - phylum "Deferribacteres" (0/1462)
    - phylum "Deinococcus-Thermus" (0/5035)
    - phylum "Dictyoglomi" (0/71)
    - phylum "Elusimicrobia" (37/782)
    - phylum "Fibrobacteres" (0/1863)
    - phylum "Fusobacteria" (1/17964)
    - phylum "Gemmatimonadetes" (1/5338)
    - phylum "Lentisphaerae" (0/2628)
    - phylum Nitrospirae (2/8171)
    - phylum "Planctomycetes" (8/31651)
    - phylum "Proteobacteria" (184478/1031919)
    - phylum "Spirochaetes" (0/30375)
    - phylum "Synergistetes" (2/2996)
    - phylum "Tenericutes" (0/10618)
    - phylum "Thermodesulfobacteria" (0/249)
    - phylum "Thermotogae" (14/1199)
    - phylum BRC1 (0/628)
    - phylum Parcubacteria (0/942)
    - phylum Microgenomates (0/322)
    - phylum SR1 (6/1084)
    - phylum Candidatus Saccharibacteria (1/7052)
    - phylum Latescibacteria (0/1162)
    - phylum "Armatimonadetes" (1/3243)
    - phylum "Verrucomicrobia" (13/27439)
    - phylum "Acidobacteria" (576/74631)
    - phylum Firmicutes (99/811625)
    - phylum Cyanobacteria/Chloroplast (11/70404)
    - phylum Marinimicrobia (3/1637)
    - phylum Aminicenantes (0/2296)
    - phylum Omnitrophica (0/38)
    - phylum Acetothermia (0/89)
    - phylum Poribacteria (0/354)
    - phylum Atribacteria (0/168)
    - phylum Cloacimonetes (1/11224)
    - phylum Candidatus Calescamantes (0/5)
    - phylum candidate division WPS-1 (110/1224)
    - phylum candidate division WPS-2 (0/376)
    - phylum Hydrogenedentes (51/985)
    - phylum candidate division ZB3 (0/95)
    - phylum Ignavibacteriae (0/2130)
    - phylum Nitrospinae (0/821)
    - Archaea Outgroup (0/1)
  - ▶ unclassified_Bacteria (647/111907)
  - domain Archaea (0/154357)
  - domain Fungi (0/108901)
  - [illegible]

| Nso190 | 5’-CGATCCCCTGCTTTTCTC -3’ |
|---|---|

**Lineage** *(click node to return it to hierarchy view)*:

Root (1398/3333501) ; Bacteria (1398/3070243) ; "Proteobacteria" (1394/1031919) ; Betaproteobacteria (1387/217518)

**Hierarchy View:**

order Nitrosomonadales (1295/7530) (hits/total searched) [list results for this node]
- family **Nitrosomonadaceae** (1291/7522)
  - ▶ genus **Nitrosomonas** (248/911)
  - ▶ genus **Nitrosospira** (993/5714)
  - ▶ **unclassified_Nitrosomonadaceae** (50/897)
- family **Spirillaceae** (4/7)
  - ▶ genus **Spirillum** (4/7)
  - unclassified_Spirillaceae (0/0)
- unclassified_Nitrosomonadales (0/1)

| Nmo218 | 5’-CGGCCGCTCCAAAGCAT -3’ |
|---|---|

**Lineage (click node to return it to hierarchy view):**

Root (1472/3333501) ; Bacteria (1472/3070243) ; "Proteobacteria" (1468/1031919) ; Betaproteobacteria (1465/217518) ; Nitrosomonadales (391/7530)

**Hierarchy View:**

family Nitrosomonadaceae (391/7522) (hits/total searched) [list results for this node]
- genus Nitrosomonas (17/911)
- genus Nitrosospira (53/5714)
- unclassified_Nitrosomonadaceae (321/897)

## Non-specific targets

**Lineage (click node to return it to hierarchy view):**

Root (1472/3333501) ; Bacteria (1472/3070243)

**Hierarchy View:**

phylum "Proteobacteria" (1468/1031919) (hits/total searched) [list results for this node]
- class Alphaproteobacteria (0/254486)
- class Betaproteobacteria (1465/217518)
  - order Burkholderiales (1004/143334)
    - family Alcaligenaceae (0/10811)
    - family Burkholderiaceae (0/30570)
    - family Comamonadaceae (1/55256)
    - family Oxalobacteraceae (996/22528)
    - family Sutterellaceae (0/3312)
    - family Burkholderiales_incertae_sedis (1/14036)
    - unclassified_Burkholderiales (6/6821)
  - order Hydrogenophilales (1/2527)
    - family Hydrogenophilaceae (1/2527)
    - unclassified_Hydrogenophilales (0/0)
  - order Methylophilales (0/6132)
  - order Neisseriales (0/20136)
  - order Nitrosomonadales (391/7530)
    - family Nitrosomonadaceae (391/7522)
    - family Spirillaceae (0/7)
    - unclassified_Nitrosomonadales (0/1)
  - order "Procabacteriales" (0/15)
  - order Rhodocyclales (1/14919)
    - family Rhodocyclaceae (1/14919)
    - unclassified_Rhodocyclales (0/0)
  - order Gallionellales (0/2444)
  - order Ferrovales (0/1301)
  - order Ferritrophicales (0/220)
  - genus Chitinivorax (0/26)
  - unclassified_Betaproteobacteria (68/18934)
- class Deltaproteobacteria (0/83856)
- class Epsilonproteobacteria (0/21128)
- class Gammaproteobacteria (0/440369)
- class "Zetaproteobacteria" (0/579)
- unclassified_"Proteobacteria" (3/13983)

* Nmo218 is not very specific but the undesired targets (e.g. Oxalobacteraceae) were not detected by amplicon sequencing to be present in the samples tested in this study.

| Nse1472 | 5’-ACC CCA GTC ATG ACC CCC -3’ |
|---|---|

**Lineage** *(click node to return it to hierarchy view):*

Root (215/3333501) ; Bacteria (215/3070243) ; "Proteobacteria" (213/1031919) ; Betaproteobacteria (211/217518)

**Hierarchy View:**

order Nitrosomonadales (209/7530) (hits/total searched) [list results for this node]
- family Nitrosomonadaceae (209/7522)
  - ▶ genus Nitrosomonas (208/911)
  - genus Nitrosospira (0/5714)
  - ▶ unclassified_Nitrosomonadaceae (1/897)
- family Spirillaceae (0/7)
- unclassified_Nitrosomonadales (0/1)

| Nsv443 | 5’-CCGTGACCGTTTCGTTCCGGC -3’ |
|---|---|

**Lineage** *(click node to return it to hierarchy view):*

Root (945/3333501) ; Bacteria (945/3070243) ; "Proteobacteria" (944/1031919) ; Betaproteobacteria (940/217518)

**Hierarchy View:**

order Nitrosomonadales (934/7530) (hits/total searched) [list results for this node]
- family Nitrosomonadaceae (934/7522)
  - genus Nitrosomonas (0/911)
  - ▶ genus Nitrosospira (934/5714)
  - unclassified_Nitrosomonadaceae (0/897)
- family Spirillaceae (0/7)
- unclassified_Nitrosomonadales (0/1)

# Primer coverage for universal primers used in HOPE to detect for total bacteria

| 27F | To be used as primer! |
|---|---|
| **Accession no.** | pB-03784 |
| **Name** (Alm et al., 1996)* | S-D-Bact-0008-d-S-20 |
| **Specificity** | Bacteria |
| **Check specificity** | probeCheck silva RDP greengenes |
| **Evaluate primer pair** | Select 2nd primer S-*-Univ-0517-a-A-15, Uni522R ▼<br>Evaluate using SILVA TestPrime |
| **Probe category** | ▶ primer |
| **Target molecule** | 16S rRNA |
| **Position*** | 6 - 25 |
| **Direction** | forward primer |
| **Sequence*** | 5'- AGA GTT TGA TCM TGG CTC AG -3' |
| **Length** [nt] | 20 |
| **MW** [g/mol] | 5854 |
| **G+C content** [%] | 45 |
| **Tm** [°C]* | 51 |
| **Coverage (full match)*** | Archaea: 0 %<br>Bacteria: 81.8 %<br>Eukaryotes: 0 % |
| **Coverage (1 mismatch)*** | Archaea: 0 %<br>Bacteria: 91.4 %<br>Eukaryotes: 0 % |
| **Reference** | Hongoh Y, Ohkuma M, Kudo T. Molecular analysis of bacterial microbiota in the gut of the termite reticulitermes speratus (isoptera; rhinotermitidae). FEMS Microbiol. Ecol. 44, 231-242 (2003).<br>▶ Abstract (PUBMED) |

| EUB338 (Bact338) (bacterial) | Tested for in situ hybridization. |
|---|---|
| **Accession no.** | pB-00159 |
| **Name** (Alm et al., 1996)* | S-D-Bact-0338-a-A-18 |
| **Specificity** | most Bacteria<br>Coverage/hits target group: 90% / 169389 (domain Bacteria )<br>Hits non-target groups: 0 |
| **Check specificity** | probeCheck silva RDP greengenes |
| **Probe category** | ▶ higher taxonomic levels |
| **Target molecule** | 16S rRNA |
| **Position*** | 338 - 355 |
| **Sequence*** | 5'- GCT GCC TCC CGT AGG AGT -3' |
| **Length** [nt] | 18 |
| **MW** [g/mol] | 5487 |
| **G+C content** [%] | 66.7 |
| **Tm** [°C]* | 55 |
| **delta Gs** [kcal/mol]* | $\Delta G_1$: -24.86; $\Delta G_2$: -1.59; $\Delta G_{12}$: -23.22 |
| **Formamide** [%]* | 0-50 |
| **Reference** | Amann R. I., Binder B. J., Olson R. J., Chisholm S. W., Devereux R. and Stahl D. A. (1990). Combination of 16S rRNA-targeted oligonucleotide probes with flow cytometry for analyzing mixed microbial populations. Appl. Environ. Microbiol. 56: 1919-1925.<br>▶ Abstract (PUBMED) |

# Primer coverage for universal primers used in amplicon sequencing to detect for total bacteria

| U515F | To be used as primer! |
|---|---|
| **Accession no.** | pB-03751 |
| **Name** (Alm et al., 1996)* | S-*-Univ-0515-a-S-19 |
| **Specificity** | Bacteria, Archaea, Eukarya |
| **Check specificity** | probeCheck silva RDP greengenes |
| **Evaluate primer pair** | Select 2nd primer S-*-Univ-0517-a-A-15, Uni522R ▼<br>Evaluate using SILVA TestPrime |
| **Probe category** | ▶ primer |
| **Target molecule** | 16S rRNA |
| **Position*** | 515 - 533 |
| **Direction** | forward primer |
| **Sequence*** | 5'- GTG CCA GCM GCC GCG GTA A -3' |
| **Length** [nt] | 19 |
| **MW** [g/mol] | 5520 |
| **G+C content** [%] | 68.4 |
| **Tm** [°C]* | 59 |
| **Coverage (full match)*** | Archaea: 54.5 %<br>Bacteria: 95.4 %<br>Eukaryotes: 92.2 % |
| **Coverage (1 mismatch)*** | Archaea: 96.2 %<br>Bacteria: 98.4 %<br>Eukaryotes: 97 % |
| **Reference** | Walters WA, Caporaso JG, Lauber CL,Berg-Lyons D, Fierer N, Knight R. PrimerProspector: de novo design and taxonomic analysis of barcoded PCR primers. Bioinformatics. 10.1093/bioinformatics/btr087 (2011).<br>▶ **Abstract (PUBMED)** |

| 907R | To be used as primer! |
|---|---|
| **Accession no.** | pB-03718 |
| **Name** (Alm et al., 1996)* | S-D-Bact-0907-a-A-20 |
| **Specificity** | Bacteria |
| **Check specificity** | probeCheck silva RDP greengenes |
| **Evaluate primer pair** | Select 2nd primer S-*-Univ-0341-b-S-17, U341F ▼<br>Evaluate using SILVA TestPrime |
| **Probe category** | ▶ primer |
| **Target molecule** | 16S rRNA |
| **Position*** | 907 - 926 |
| **Direction** | reverse primer |
| **Sequence*** | 5'- CCG TCA ATT CMT TTG AGT TT -3' |
| **Length** [nt] | 20 |
| **MW** [g/mol] | 5758 |
| **G+C content** [%] | 35 |
| **Tm** [°C]* | 47 |
| **Coverage (full match)*** | Archaea: 0 %<br>Bacteria: 89.7 %<br>Eukaryotes: 0 % |
| **Coverage (1 mismatch)*** | Archaea: 0.9 %<br>Bacteria: 96.9 %<br>Eukaryotes: 77 % |
| **Reference** | Muyzer G, A, Teske C.O, Wirsen , Jannasch H.W Phylogenetic relationships of Thiomicrospira species and their identification in deep-sea hydrothermal vent samples by denaturing gradient gel electrophoresis of 16S rDNA fragments. Arch. Microbiol. 164, 165-172 (1995).<br>▶ **Abstract (PUBMED)** |

# Nitrifying bacterial populations detected by 515F and 907R primers

## Probe Match Result

[ start over | help | options ]

Display depth: Auto

Differences Allowed: 0

Probe: 5'TTACCGCGGCKGCTGRCAC 3'
Target: 5'GTGYCAGCMGCCGCGGTAA 3'

Probe: 5'CCCCGYCAATTCMTTTRAGT 3'
Target: 5'ACTYAAAKGAATTGRCGGGG 3'

**Lineage *(click node to return it to hierarchy view)*:**

Root (1490490/3333501) ; Bacteria (1490490/3070243) ; "Proteobacteria" (483848/1031919) ; Betaproteobacteria (110367/217518)

**Hierarchy View:**

order Nitrosomonadales (4877/7530) (hits/total searched) [list results for this node]
- family Nitrosomonadaceae (4873/7522)
  - genus Nitrosomonas (401/911)
  - genus Nitrosospira (4038/5714)
  - unclassified_Nitrosomonadaceae (434/897)
- family Spirillaceae (3/7)
  - genus Spirillum (3/7)
  - unclassified_Spirillaceae (0/0)
- unclassified_Nitrosomonadales (1/1)

**Lineage *(click node to return it to hierarchy view)*:**

Root (1490490/3333501) ; Bacteria (1490490/3070243)

**Hierarchy View:**

phylum Nitrospirae (2505/8171) (hits/total searched) [list results for this node]
- class "Nitrospira" (2505/8171)
  - order "Nitrospirales" (2505/8171)
    - family "Nitrospiraceae" (2505/8171)
      - genus Leptospirillum (558/2520)
      - genus Nitrospira (1832/5378)
      - genus Thermodesulfovibrio (87/230)
      - unclassified_"Nitrospiraceae" (28/43)
    - unclassified_"Nitrospirales" (0/0)
  - unclassified_"Nitrospira" (0/0)
- unclassified_Nitrospirae (0/0)

# Nitrifying bacterial populations detected by 515F and 907R primers

## Probe Match Result

[ start over | help | options ]

Display depth: Auto ▼

Differences Allowed: 0 ▼

Probe: 5'TTACCGCGGCKGCTGRCAC 3'
Target: 5'GTGYCAGCMGCCGCGGTAA 3'

Probe: 5'CCCCGYCAATTCMTTTRAGT 3'
Target: 5'ACTYAAAKGAATTGRCGGGG 3'

**Lineage *(click node to return it to hierarchy view)*:**

Root (1490490/3333501) ; Bacteria (1490490/3070243) ; "Proteobacteria" (483848/1031919) ; Alphaproteobacteria (109223/254486) ; Rhizobiales (37023/84204) ; Bradyrhizobiaceae (6309/13197)

**Hierarchy View:**

▼ genus Nitrobacter (113/195) (hits/total searched) [list results for this node]

* S000269985 Nitrobacter sp. PBAB17; AY508476
* S000392359 Nitrobacter winogradskyi; IFO 14297; AF363288

S000414380 Nitrobacter winogradskyi; W; L11661
S000414381 Nitrobacter sp.; LL; L11662
S000414382 Nitrobacter hamburgensis (T); X14; L11663
S000414627 Nitrobacter hamburgensis; Nb14; L35502
S000414631 Nitrobacter winogradskyi; L35506
S000414632 Nitrobacter winogradskyi; L35507
S000414636 Nitrobacter sp.; R6; L35511
S000428396 Nitrobacter alkalicus (T); AN1; AF069956
S000428397 Nitrobacter alkalicus; AN2; AF069957
S000428398 Nitrobacter alkalicus; AN4; AF069958
S000434954 Nitrobacter winogradskyi; ATCC 14123; AY055796
S000434955 Nitrobacter sp. NRB5220; AY055797
S000492976 uncultured alpha proteobacterium; AKYH732; AY921742
S000535828 uncultured Nitrobacter sp.; Nbac20; AY876604
S000535829 uncultured Nitrobacter sp.; Nbac20-3-12s; AY876605
